# Functional sensory symptoms and signs: a case-control study of 102 patients

**DOI:** 10.1093/braincomms/fcag031

**Published:** 2026-02-03

**Authors:** Glenn Nielsen, Rory Higgins, Jon Stone, Jan Coebergh, Mark J Edwards

**Affiliations:** Neuroscience and Cell Biology Research Institute, City St George’s, University of London, London SW17 0RE, UK; Neuroscience and Cell Biology Research Institute, City St George’s, University of London, London SW17 0RE, UK; Toronto Rehabilitation Institute, University Health Network, Toronto, ON M5G 2A2, Canada; Centre for Clinical Brain Sciences, Royal Infirmary of Edinburgh, Edinburgh EH16 4SB, UK; Department of Neurology, Ashford St.Peter’s Hospitals NHS Foundation Trust, Chertsey KT16 0PZ, UK; Department of Neurology, St.George’s Hospital NHS Foundation Trust, London SW17 0QT, UK; Department of Basic & Clinical Neuroscience, Institute of Psychiatry, Psychology & Neuroscience, King’s College London, London SE5 8AB, UK; Department of Neuropsychiatry, South London and Maudsley NHS Foundation Trust, London SE5 8AZ, UK

**Keywords:** functional neurological disorder, FND, sensation, stroke

## Abstract

Despite being frequently reported by patients, the prevalence, character and clinical relevance of sensory symptoms in functional neurological disorder (FND) is unknown. This study aimed to (i) estimate the frequency and explore the characteristics of sensory symptoms and signs (excluding the special senses) in patients with motor-FND, (ii) compare these features to patients with recent stroke, and (iii) investigate potential mechanisms underlying functional sensory symptoms. In this prospective observational cohort study, 102 patients with motor-FND and 75 patients with recent stroke were assessed using structured clinical interviews, body maps, validated questionnaires, clinical assessment and quantitative sensory testing. Motor-FND participants were followed up at 12-months. Data were analysed using thematic analysis for symptom description, descriptive statistics for frequency and regression models to explore predictors of symptom severity and change. Sensory symptoms were highly prevalent in motor-FND, reported by 96% compared to 67% of mild to moderate recent stroke. However, 27% of motor-FND and 36% of stroke only endorsed experiencing sensory symptoms after prompting. Motor-FND participants described a broader spectrum of sensory experiences compared to stroke, including numbness, paraesthesia, movement-related perceptions and abstract descriptions. Feelings of limb absence/‘feels dead’ (19% versus 1%) and areas of complete sensory absence (27% versus 15%) were more commonly reported in individuals with FND than in mild-moderate stroke. The motor-FND group experienced pain more frequently than the stroke group (88% versus 41%) and more frequently endorsed having a high pain tolerance (70% versus 49%). The distribution of sensory symptoms differed from the distribution of pain. Sensory symptoms were often perceived as severe and associated with disability and depression. Conflation of the concepts of weakness and numbness was common in both groups (21% of motor-FND versus 10% of stroke). Only one-third of motor-FND patients reported improvement in sensory symptoms at 12 months. Dissociation, body perceptual disturbance and sensory hypersensitivity were significantly more common in motor-FND. Dense midline splitting of light touch or splitting of vibration sense across the forehead or sternum were uncommon and had poor diagnostic specificity, but asymmetries in vibration sense were more common in motor-FND. Quantitative sensory testing provided no clear added diagnostic value. Sensory symptoms in motor-FND vary in nature, are highly prevalent, persistent, clinically significant and often linked to broader illness burden and psychological distress. Sensory symptoms should be routinely assessed in FND, both for diagnosis and treatment planning. Future research should evaluate targeted interventions to specifically address sensory symptoms within multidisciplinary rehabilitation frameworks.

## Teaser

Nielsen *et al.* found that sensory symptoms were highly prevalent, diverse and disabling in patients with functional motor disorder compared to stroke. Sensory symptoms persisted at 12-months and correlated with disability and depression. Classic sensory signs showed limited diagnostic utility. Findings highlight sensory symptoms as a core feature of functional neurological disorder.

## Graphical Abstract

**Graphical Abstract:**
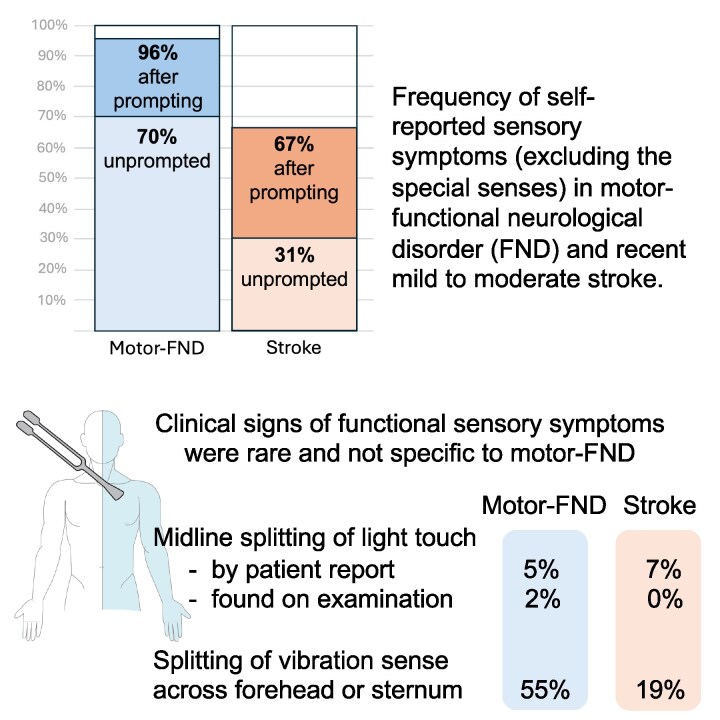

## Introduction

The resurgence of interest in functional neurological disorder (FND) has resulted in a detailed reappraisal of clinical phenomenology. This has encompassed re-discovery of insights from the past, the application of modern scientific methods, larger cohorts to confirm or sometimes refute the sensitivity and specificity of classic symptoms and signs and the delineation of new clinical features and syndromes within the broad spectrum of FND.^1,2^ Compared to motor and other presentations of FND, sensory symptoms have received less attention in this reappraisal, despite being amongst the most commonly reported symptoms in FND.^3^ Sensory symptoms were once considered a core, even essential, feature of FND,^4^ however, they rarely feature in the current literature. Sensory symptoms have been excluded from recent review papers,^1^ and a topic review from 2016 emphasized that many clinical insights still have their origin in historical literature.^5^ We currently lack data on the prevalence, impact and prognosis of sensory symptoms in FND. Lack of research interest may relate to their subjective nature and perhaps a perception from health professionals that they have limited impact. Additionally, they currently have a limited role in making a ‘rule-in’ diagnosis,^6^ which may have disincentivized interest.

The primary aim of this research was to explore the frequency and clinical features of sensory symptoms, not including pain and abnormalities in the special senses, in patients diagnosed with FND presenting with motor symptoms (motor-FND) in comparison to those with recent stroke. Secondary aims included evaluating methods to assess functional sensory symptoms and exploring potential mechanisms. We adopted the conventional clinical definition of symptom, that is, a patient-reported subjective experience, and the term *sensory disturbance* to describe changes on examination.^7^

## Materials and methods

### Study design

We conducted a prospective observational cohort study of sensory symptoms and related experiences in patients with motor-FND. A cohort of patients with recent stroke was concurrently recruited to serve as a contextual comparison group. The motor-FND group was only were followed up at 12-months by telephone. Ethical approval was obtained by the London Bloomsbury Research Ethics Committee (reference 21/LO/0724, 12 November 2021). All participants gave informed consent to participate.

### Participants and setting

#### Motor-FND group

People attending specialist FND neurology clinics at a tertiary hospital in London, United Kingdom, over the period from 1 December 2021 until 8 August 2024 were screened for recruitment. The inclusion criteria were: (i) a ‘clinically definite’ diagnosis of motor-FND^8^; and (ii) aged 18 or over. Exclusion criteria were: (i) another diagnosis that is likely to explain the presenting sensory symptoms; (ii) severe psychiatric comorbidity; (iii) unresolved compensation claims or litigation; (iv) unable to understand English sufficiently to complete the study questionnaires; and (v) a documented learning disability or incapacity to give informed consent.

#### Stroke group

A stroke control group was recruited from inpatients at the same institution from 1 December 2021 to 9 September 2024. The inclusion criteria were: (i) aged 18 or over; (ii) CT or MRI confirmed diagnosis of stroke in the past 6 months; and (iii) documented motor signs related to the stroke. Exclusion criteria were the same as for the motor-FND group, with the following additions: (i) unilateral spatial neglect; (ii) cognitive or communication deficits that would prevent questionnaire completion; (iii) unstable physiological state or deterioration in medical condition in the previous 24 h; and (iv) documented palliative care pathway.

### Procedures

Eligible participants were told about the study and provided with an information sheet. Those consenting to be contacted were screened for eligibility and invited to participate in the study. Assessments were conducted face-to-face by author RH in an outpatient hospital clinic room or at the bedside. Clinical and demographic data (listed in Table 1) were extracted from the medical records.

**Table 1:** Demographic and clinical characteristics

	Motor-FND, *n* = 102	Stroke, *n* = 75
Age, mean (SD)	43.8 (14.1)	61.7 (12.9)
Age, range	20–88	28–90
Female	81 (79.4%)	36 (48.0%)
Male	20 (19.6%)	39 (52.0%)
Nonbinary	1 (1.0%)	0
Ethnicity		
White	88 (86.3%)	49 (65.3%)
Asian	5 (4.9%)	9 (12.0%)
Black	6 (5.9%)	14 (18.7%)
Arab	1 (1.0%)	2 (2.7%)
Mixed	2 (2.0%)	1 (1.3%)
Employed or studying	45 (44.1%)	35 (47.3%)
Home-maker	7 (6.9%)	2 (2.7%)
Unemployed	19 (18.6%)	13 (17.6%)
Retired (unrelated to health)	7 (6.9%)	19 (25.7%)
Not working due to ill health	24 (23.5%)	5 (6.8%)
Missing data	0	1
Years of schooling, mean (SD)	15.3 (2.9)	13.9 (2.8)
Symptom duration, median (IQR)	4.4 (2.2, 8.2) years	3 (2, 6) days since stroke
Motor-FND, dominant motor symptom/presentation		
Weakness	25/102 (24.5%)	
Gait disturbance	23/102 (22.5%)	
Tremor	10/102 (9.8%)	
Dystonia	9/102 (8.8%)	
Jerks	2/102 (2.0%)	
Mixed movement disorders	33/102 (32.5%)	
Stroke mechanism		
Infarct		60/75 (80.0%)
Haemorrhage		13/75 (17.3%)
Both		2/75 (2.7%)
National Institute of Health (NIH) Stroke Scale, median (IQR)		6.0 (4.0, 8.75)
Laterality of stroke symptoms		
Left sided symptoms		42/75 (56.0%)
Right sided symptoms		31/75 (41.3%)
Both sides similarly affected		2/75 (2.7%)
Domains from the RAND 36-Item Health Survey^a^		
Physical functioning (score range 0–100), mean (SD)	35.7 (20.8)	24.6 (25.5)
Energy/fatigue domain (score range 0–100), mean (SD)	26.2 (20.7)	47.6 (20.1)
Frequency endorsing, ‘Do you experience regular pain?’	89/101 (88.1%)	30/74 (40.5%)
Pain intensity over the last week (VAS 0–10), median (IQR)	5 (4, 7)	3 (0, 6)

^a^Higher scores represent better health.

Abbreviations: VAS = visual analogue scale.

#### Questionnaires

Participants completed two items from the RAND 36-Item Health Survey (physical functioning and fatigue)^9,10^; pain visual analogue scale (VAS)^11^; Patient Health Questionnaire-9 depression scale (PHQ-9)^12^; Generalized Anxiety Disorder-7 (GAD-7)^13^; Patient Health Questionnaire-15 for somatic symptom severity (PHQ-15)^14^; and participant perception of sensory symptom severity and motor symptom severity (7-point ordinal scales). The motor-FND group repeated the questionnaires at 12-month follow-up, with an additional question asking them to rate the change in severity of their sensory symptoms (7-point Likert scale ranging from ‘very much worse’ to ‘very much improved’). The stroke group completed only a subset of the questionnaires (first three listed), as not all questionnaires were appropriate for the experience of acute stroke.

#### Semi-structured clinical interview

Semi-structured clinical interviews explored the experience and perceptions of sensory symptoms. To minimize suggestion, we started the interview by asking participants to list their symptoms in order of importance. If participants did not list sensory symptoms, they were prompted with the question, ‘Do you experience problems with sensation?’. We explored if participants understood the difference between the concepts of weakness and numbness by asking them to endorse one of three options: (i) I feel confident I understand the difference; (ii) I think I understand the difference; or (iii) I don’t understand the difference. After attempting to clarify the different concepts, sensory, motor and pain symptoms were mapped on body charts, and details of each symptom were discussed. The interview included the following validated questionnaires: the revised Bath Body Perception Disturbances Questionnaire (the revised scale excludes an item related to attention due to lack of internal consistency)^15^; the Peritraumatic Dissociative Experiences Questionnaire^16^; items from dissociation scales^17-19^; and we screened for symptoms of a panic attack at FND symptom onset according to the Diagnostic and Statistical Manual of Mental Disorders (DSM-5 TR) criteria.^20^ Further interview questions were informed by historical texts on FND and theoretical pathophysiological mechanisms for FND.^2,4,21-23^ Interviews were audio recorded.

#### Quantitative sensory testing (QST)

Mechanical detection threshold (MDT), vibration detection threshold (VDT) and pain pressure threshold (PPT) assessments were conducted. The procedure was informed by the equipment instruction manuals and published recommendations for QST.^24,25^ Semmes-Weinstein Monofilaments (The Touch-Test^TM^, North Coast Medical, 20 filament kit) were used to measure MDT. The findings are interpreted according to published threshold categories of sensory disturbance.^26^ A Rydel-Seiffer tuning fork (64 Hz) was used to measure VDT^27,28^ and assess for midline splitting of vibration sense.^6^ A pressure algometer (Wagner Instruments Pain Test^TM^) was used to measure PPT, which were reported in units N/cm^2^. Hypersensitivity and hyposensitivity in motor-FND compared to stroke was explored by comparing PPT 25th and 75th percentiles respectively.^29^

#### Physical assessment

A physical examination was conducted that included assessment of sensory impairment boundaries and signs of functional sensory and motor symptoms, including midline splitting of light touch, splitting of vibration sense and Hoover’s sign for functional leg weakness, according to published examination techniques.^6^

Additional details of the study methods, outcome measures, the interview topic guide and QST procedure can be found in the Supplementary material.

### Statistical analysis

The sample size was calculated to determine the frequency of sensory symptoms in people with motor-FND. Based on an expected frequency of 65%,^30^ 95% level of confidence and a precision of 10%, the required sample size was 88.^31^ The sample size target was rounded up to 100 to account for dropouts and missing data. We aimed to recruit an equivalent-sized sample of people with acute stroke. Descriptive statistics are reported with means (SD), medians (IQR) or frequencies (%).

Categories of sensory symptoms were derived through an inductive qualitative content analysis of participant descriptions.^32^ Words used to describe sensory symptoms were extracted from the clinical interviews for both motor-FND and stroke groups (by author RH). Two authors (R.H. and G.N.) grouped similar descriptors together, descriptors were grouped into themes and similar themes were grouped into broader categories. At each stage in the analysis, the themes and categories were cross-referenced with the original interview data and discussed with the wider research team. The themes and categories were updated until there was agreement amongst the research team.

For the motor-FND group, associations between self-reported sensory symptom severity (7-point ordinal scale) and clinical measures (RAND physical functioning, RAND fatigue, pain VAS, PHQ-9, GAD-7 and PHQ-15) were examined using Spearman’s rank correlation coefficients. Variables significantly correlated with sensory symptom severity in the univariable analysis were entered into a multivariable ordinal logistic regression model using logit link function to identify variables independently associated with sensory symptom severity. Odds ratios and 95% confidence intervals were calculated for variables that were statistically significant in the multivariable model.

Predictors of perception of change in sensory symptom severity at 12-months were explored from baseline outcome measures using ordinal regression modelling. To determine if motor and sensory symptoms improve or deteriorate in tandem, the association between reported change in sensory symptom severity and change in motor symptoms was explored. In this analysis, motor symptoms severity was dichotomized into improved from baseline versus no change or worse and the association with sensory symptom change (7-point ordinal scale) was analysed using logistic regression. Additionally, ordinal regression modelling was used to assess the association between reported change in sensory symptom severity and RAND physical functioning scores at follow-up.

Group differences were compared using chi-square or Fisher’s exact test for categorical variables, and Mann–Whitney *U* test for continuous variables that were not normally distributed. *P*-values are reported as exploratory, acknowledging the limitations of multiple hypothesis testing.

All statistical analyses were performed using SPSS 29.0. Due to low levels of missing data, we conducted complete case analyses.

## Results

Reporting guidelines for observational cohort studies are followed.^33^

### Participants

The final sample includes 102 people with motor-FND and 75 with recent stroke. See Table 1 for demographic and clinical characteristics. The motor-FND group was classified according to the dominant motor presentation. These were weakness 25/102 (24.5%), gait disturbance 23/102 (22.5%), tremor 10/102 (9.8%), dystonia 9/102 (8.8%), jerks 2/102 (2.0%) and mixed movement disorders 33/102 (32.5%). In addition, 26/101 (25.7%) experienced functional symptoms of speech, and 13/101 (12.9%) reported experiencing functional seizures. Among the stroke group, 60/75 (80.0%) were classified as an infarct, 13/75 (17.3%) a haemorrhage and 2/75 (2.7%) were reported as both. Regarding stroke interventions, 9/75 (12.0%) underwent thrombectomy and 6/75 (8.0%) received thrombolysis. The interquartile range of the National Institute of Health (NIH) Stroke Scale scores corresponded with the lower range of moderate stroke severity (median 6, IQR 4.0, 8.75, and moderate range has been reported as 5–15).^34^ Laterality of stroke symptoms was right sided 31/75 (41.3%), left sided 42/75 (56.0%) and both sides were similarly affected in 2/75 (2.7%). Further information on the stroke group can be found in the Supplementary material.

Each participant was screened for potential causes of sensory disturbance other than FND or stroke. In each case, coexisting health problems were considered not to explain the range of sensory symptoms reported by the participant. See Supplementary material for more details.

### Frequency of sensory symptoms in motor-FND and stroke

When participants were asked to list all their neurological symptoms without prompting, 71/102 (69.6%) of the motor-FND group and 23/75 (30.7%) of the stroke group reported experiencing sensory symptoms.

If sensory symptoms were not reported unprompted, participants were asked if they experienced them. Once prompted, 98/102 (96.1%) of the motor-FND group and 50/75 (66.7%) of the stroke group endorsed experiencing sensory symptoms (26.5% of motor-FND and 36.0% of stroke only endorsed sensory symptoms after prompting).

Sensory symptoms were reported as the worst symptom by 17/102 (16.7%) participants with motor-FND and 10/75 (13.3%) with stroke. Sensory symptoms ranked among the top three worst symptoms for 44/102 (43.1%) motor-FND participants and 18/75 (24.0%) stroke participants.

**Figure 1:**
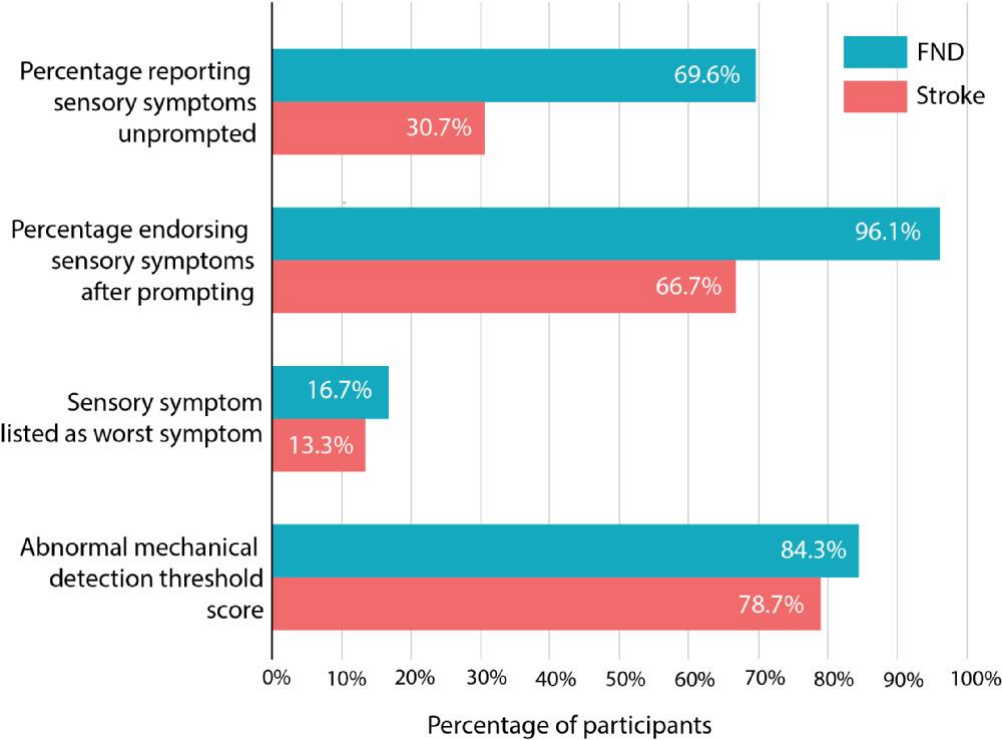
Frequency of sensory symptoms amongst people with functional neurological disorder motor symptoms (*n* = 102) and mild to moderate stroke (*n* = 75).

The frequency of sensory disturbance was considered from the perspective of the number who had abnormal QST results based on published thresholds^26^; 86/102 (84.3%) of the motor-FND group and 59/75 (78.7%) of the stroke group had at least one site with an abnormal QST score. See Fig. 1.

### Sensory symptom severity rating

The motor-FND group rated the severity of their sensory symptoms on a seven-point ordinal scale. No sensory symptoms, borderline or mild symptoms were reported by 28/101 (27.7%); moderate or marked symptoms were reported by 40/101 (39.6%); and severe or extreme symptoms were reported by 45/101 (44.5%). See Table 2 for breakdown and category qualifiers.

**Table 2:** Clinical outcomes, motor-FND group

	Motor-FND, max *n* = 101	
Patient Health Questionnaire-9 (PHQ-9, depression scale), score range 0–27		
Mean (SD)	12.8 (6.4)	
N scoring 10 or above (cut-off score for cases of depression)	69/101 (68.3%)	
Generalized Anxiety Disorder-7 (GAD-7), score range 0–21		
Mean (SD)	9.5 (5.9)	
N scoring 10 or above (cut-off score for cases of anxiety)	51/101 (50.5%)	
Patient Health Questionnaire-15 (PHQ-15), score range 0–30		
Mean (SD)	12.3 (4.8)	
Sensory and Motor Symptom Severity (7-point ordinal scale)	**Sensory**	**Motor**
No symptoms^a^	4/101 (4.0%)	2/101 (2.0%)
Borderline (subtle or very minor impact)	5/101 (5.0%)	3/101 (3.0%)
Mild (causing problems occasionally)	19/101 (18.8%)	5/101 (5.0%)
Moderate (causing difficulties a few days in the week)	25/101 (24.8%)	22/101 (21.8%)
Marked (interfered with social/school/work most days)	15/101 (14.9%)	24/101 (23.8%)
Severe (resulting in needing help from others for daily activities)	23/101 (22.8%)	32/101 (31.7%)
Extreme (result in needing hospitalization or nursing care)	10/101 (9.9%)	13/101 (12.9%)
12-month follow-up: Compared to one year ago, my sensory symptoms (numbness, tingling, pins and needles, etc.) are:		
Very much worse	7/90 (7.8%)	
Much worse	7/90 (7.8%)	
Minimally worse	17/90 (18.9%)	
No change	32/90 (35.6%)	
Minimally improved	16/90 (17.8%)	
Much improved	8/90 (8.9%)	
Very much improved	3/90 (3.3%)	
Total reporting no change or worse	63/90 (70.0%)	
Total reporting improvement	27/90 (30.0%)	

^a^All participants had motor symptoms according to the clinician screening for eligibility to participate. In this patient-reported data, 2/101 FND group participants reported not having motor symptoms.

### Categories of sensory experiences based on subjective descriptions

For the motor-FND group, 277 descriptions of sensory symptoms were extracted from the interviews of all 96 participants reporting sensory symptoms and 77 descriptions were extracted from the 50 stroke group participants reporting sensory symptoms. The descriptors were classified into six groups (Table 3), further details of this process are described in the Supplementary material.

**Table 3:** Clinical findings

	Motor-FND	Stroke	*P* value^b^
Frequency of sensory disturbance category			
Numbness/reduced sensation	69/102 (67.6%)	41/75 (54.7%)	0.079
Paraesthesia	68/102 (66.7%)	15/75 (20.0%)	**<0**.**001**
Dead or absent	20/102 (19.6%)	1/75 (1.3%)	**<0**.**001**
Muscle and movement related sensory experiences	11/102 (10.8%)	2/75 (2.7%)	0.041
Abstract sensory experiences^a^	23/102 (22.5%)	3/75 (4.0%)	**<0**.**001**
Complete sensory loss/absent sensation	27/102 (26.5%)	8/75 (10.7%)	0.103
Area of sensory absence experienced intermittently	15/102 (14.7%)	0	**<0**.**001**
Other characteristics of sensory symptoms			
Unique sensory symptoms within individuals, median (IQR)	2 (1, 3)	1 (0, 2)	**<0**.**001**
Three or more unique sensory symptoms	24/96 (25.0%)	3/50 (6.0%)	0.005
Experiences an intermittent sensory symptom	74/96 (77.1%)	18/50 (36.0%)	**<0**.**001**
Only has intermittent sensory symptoms	45/96 (46.9%)	14/50 (28.0%)	0.027
Laterality of sensory disturbance, by self-report			
Central or both sides affected	56/102 (54.9%)	8/75 (10.7%)	**<0**.**001**
Left sided	25/102 (24.5%)	24/75 (32.0%)	0.271
Right sided	17/102 (16.7%)	18/75 (24.0%)	0.226
No sensory disturbance reported	4/102 (3.9%)	25/75 (33.3%)	**<0**.**001**
Hypersensitivity, by self-report			
Skin	53/100 (53.0%)	10/73 (13.7%)	**<0**.**001**
Light (photophobia)	63/100 (63.0%)	15/73 (20.5%)	**<0**.**001**
Sound	65/100 (65.0%)	11/73 (15.1%)	**<0**.**001**
Smell	40/100 (40.0%)	6/73 (8.2%)	**<0**.**001**
Taste	20/100 (20.0%)	9/73 (12.3%)	0.182
Movement	40/100 (40.0%)	13/73 (17.8%)	0.002
Sensory disturbance of internal surfaces			
Opening bowels	22/100 (22.0%)	4/73 (5.5%)	0.003
When passing urine	28/100 (28.0%)	2/73 (2.7%)	**<0**.**001**
Inside mouth	25/100 (25.0%)	17/73 (23.3%)	0.795
Inside nose	13/100 (13.0%)	8/73 (11.0%)	0.685
Surface of eyes	26/100 (26.0%)	9/73 (12.3%)	0.027
Saddle-area sensory disturbance	36/100 (36.0%)	8/73 (11.0%)	**<0**.**001**
Endorsing suffering with cold hands or feet (all participants)	82/99 (82.8%)	28/69 (40.6%)	**<0**.**001**
Females only	71/80 (88.8%)	14/36 (38.9%)	**<0**.**001**
After completing the assessments today, has your opinion on the severity of your sensory symptoms changed?’			
Worse than I previously thought	13/99 (13.1%)	3/71 (4.2%)	0.050
The same, no change	66/99 (66.7%)	55/71 (77.5%)	0.125
Better than I previously thought	20/99 (20.2%)	13/71 (18.3%)	0.758
Some people believe that focusing in on a symptom can make it worse, do you think that has happened today?			
Yes	36/93 (38.7%)	5/65 (7.7%)	**<0**.**001**
No	57/93 (61.3%)	60/65 (92.3%)	

^a^The abstract category of sensory symptoms included feelings of tightness/pressure, internal vibrations, electricity moving through the body, pulses and altered thermal regulation. Within this group, there were unusually detailed descriptions, such as, ‘feels like a tube has replaced my femur’, ‘rainfall inside my body’, and ‘I can feel my brain move when I turn’.

^b^Significance testing was performed using chi-square tests for categorical variables and Mann–Whitney *U* test for continuous variables. *P*-values that remain significant after Bonferroni correction (threshold 0.05/32 = 0.00156) are shown in bold.

#### Numbness or reduced sensation

The term numbness was mostly used to describe reduced sensation. This group included descriptors ‘dull’, ‘muted’, ‘loss’, ‘less than’ and ‘as if my hand is covered with a plastic film’. The term numbness was sometimes used to describe reduced sensation combined with paraesthesia. It was experienced by 69/102 (67.6%) of the motor-FND group and 41/75 (54.7.0%) of the stroke group.

#### Paraesthesia

This describes *positive* sensory phenomena that could suggest somatosensory pathway dysfunction.^35^ It included descriptors ‘pins and needles’, ‘tingling’, ‘prickling’, ‘burning’ and ‘hypersensitive’. It was experienced by 68/102 (66.7%) of the motor-FND group and 15/75 (20.0%) of the stroke group.

#### Dead or absent

This included descriptors ‘it feels dead’, ‘dead weight’ and ‘nothing there’. It was experienced by 20/102 (19.6%) of the motor-FND group. There was one potential example of a dead or absent sensory symptom in the stroke group (1/75, 1.3%). The participant described both lower limbs as feeling as if they belonged to someone else.

#### Muscle and movement-related experiences

These experiences were interpreted by the participants as sensory rather than motor symptoms. Descriptors included feeling ‘stiff’, ‘tired’ and ‘pulled’. It was experienced by 11/102 (10.8%) of the motor-FND group and 2/75 (2.7%) of the stroke group.

#### Abstract descriptive experiences

This category grouped together *other* experiences not typically associated with primary somatosensory pathway dysfunction. It included feelings of tightness/pressure, internal vibrations, electricity moving through the body, pulses and altered thermal regulation. Within this group there were unusual, detailed descriptions, such as, ‘feels like a tube has replaced my femur’, ‘rainfall inside my body’ and ‘I can feel my brain move when I turn’. It was experienced by 30/102 (29.4%) of the motor-FND group and 4/75 (5.3%) of the stroke group.

#### Complete sensory loss

The complete absence of sensation in an area was experienced by 27/102 (26.5%) of the motor-FND group. The loss was experienced intermittently for 15/102 (14.7%). Stroke participants with a complete loss were 8/75 (10.7%). None had an area of complete loss that was experienced intermittently.

Two participants in the motor-FND group and two in the stroke group were only able to describe their sensory symptoms as ‘different’, ‘not normal’ or ‘I don’t know’.

#### Other categorizations of sensory symptoms

The motor-FND group more frequently reported more than one unique sensory symptom and were more likely to report intermittent sensory symptoms. The motor-FND group more frequently endorsed experiencing sensory hypersensitivity to touch, light and sound. See Table 3.

### Understanding of the concept of numbness versus weakness

Self-reported understanding was similar in both groups; 51/100 (51.0%) of motor-FND and 38/73 (52.1%) of stroke were confident that they understood the difference between the concepts of weakness and numbness; 34/100 (34.0%) of motor-FND and 25/73 (34.2%) of stroke were less sure and only ‘thought’ they understood the difference; while 15/100 (15.0%) of motor-FND and 10/73 (13.7%) of stroke reported a lack of understanding of the difference.

The researcher’s assessment of the participant’s understanding, a perception based on the entire clinical interview, was similar in that approximately half appeared to recognize the difference between weakness and numbness (52/99, 52.5% of motor-FND, 34/73, 46.6% of stroke). Participants appearing to have a limited understanding of the difference were 26/99 (26.3%) of motor-FND and 32/73 (43.8%) of stroke; whereas 21/99 (21.2%) of motor-FND and 7/73 (9.6%) of stroke appeared to conflate the terms or use them interchangeably.

### Clinical presentations

**Figure 2:**
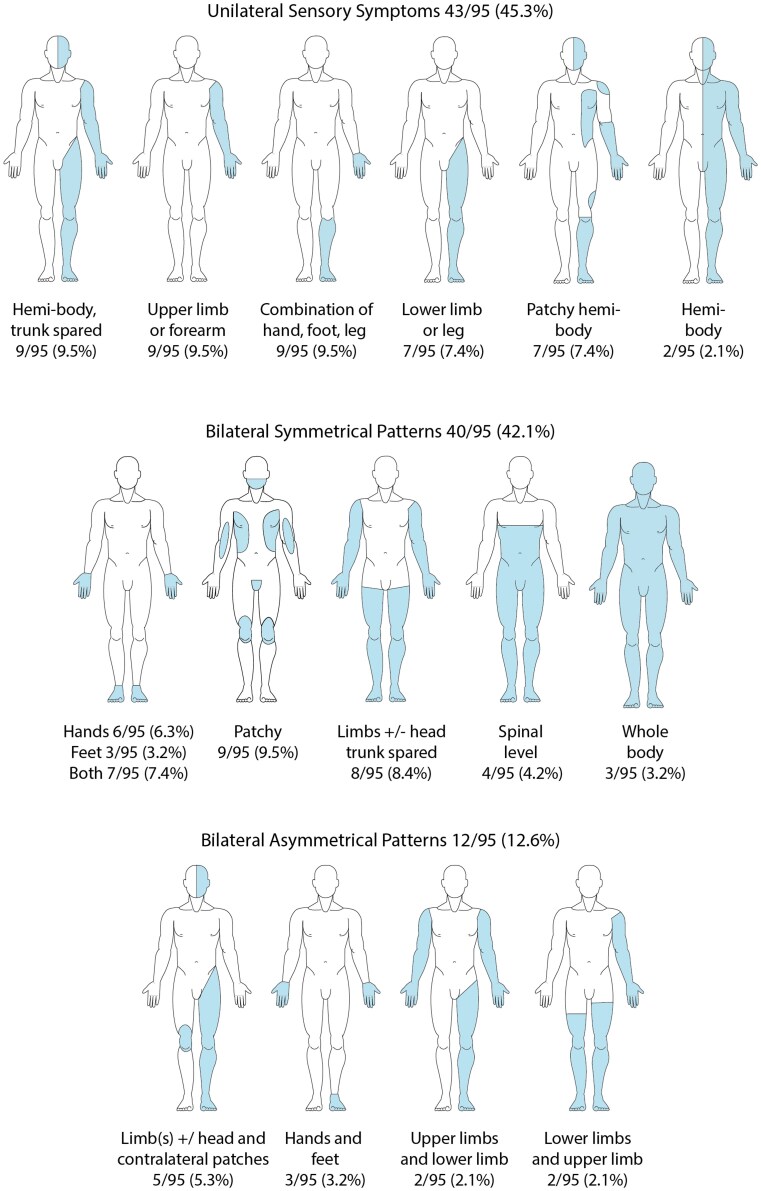
**Common patterns of sensory symptom presentation.** The figures represent common patterns. Unilateral sensory symptoms are represented on the left side for illustrative purposes only. Transition between areas and impaired sensation may be either sharply demarcated or indistinct. Other patterns not represented here include sensory impairment in the ulnar side of the hand and patchy facial sensory symptoms. The nature of the sensory symptoms spanned all reported experiences including complete loss, reduced sensation, tingling and pulsating sensations.

Body maps of sensory symptoms amongst participants with motor-FND were categorized into three groups (see Fig. 2).

- unilateral symptoms, 45% of participants with motor-FND sensory symptoms.
- bilateral symmetrical distribution, 42% of motor-FND sensory symptoms.
- bilateral and asymmetrical distribution, 13% of motor-FND sensory symptoms.

Bilateral sensory symptoms were most common (56/102, 54.9%), followed by left-sided sensory symptoms (25/102, 24.5%) and right-sided (17/102, 16.7%).

The classically described ‘glove and stocking’ pattern of sensory loss, where the limb is uniformly affected with a demarcation at or near a joint line, was seen in the motor-FND group in 20/102 (19.6%) individuals; 13/102 (12.7%) had upper limb glove patterns; 9/102 (8.8%) had lower limb stocking patterns; and there were two cases with individual fingers affected. There were only 2/75 (2.7%) participants with glove and stocking sensory disturbance in the stroke group (neither had other signs of FND found during the assessment).

Limbs with sensory symptoms were most commonly also affected by motor symptoms. For example, in symptomatic right lower limbs (*n* = 70), 77% reported both, 17% reported only motor and 6% reported only sensory symptoms. In symptomatic right upper limbs (*n* = 65), 70% reported both, 12% reported only motor and 17% reported only sensory symptoms. There was no observable or statistical relationship between FND-motor symptom type (weakness, tremor, dystonia, etc.) and the distribution of sensory symptoms.

It was more common for the motor-FND group compared to the stroke group to experience sensory symptoms affecting internal surfaces, for example, reduced sensation when passing urine (28.0% motor-FND versus 2.7% stroke). Sensory symptoms on the inside of the mouth were similar between groups (25.0% motor-FND versus 23.3% stroke), see Table 3.

We asked the motor-FND group if their sensory symptoms had led to accidental burns, cuts or injuries, to which 53/95 (55.8%) responded affirmatively.

### Clinical signs of sensory disturbance

#### Midline splitting of sensation (light touch)

Midline splitting with reduced or altered sensation on one side was subjectively described by 5/102 (4.9%) of the motor-FND group. In three cases, the whole body was involved, the left upper quadrant in one case and the face only in another case. When examined by a clinician, only in 2/102 (2.0%) cases was there an observable sharp midline demarcation in sensory disturbance on sensory testing.

Midline splitting was reported by 7/75 (9.3%) of the stroke group. In each case, the sensory impairment was described as extending along the face and the whole trunk. Two of these cases also presented with a positive Hoover’s sign for functional weakness, and therefore FND is a possible explanation for the midline splitting in these individuals. When all stroke group participants with a positive Hoover’s sign (*n* = 8) were removed from the data, 5/67 (7.5%) described midline split of light touch. On clinical examination, no stroke participants had a sharp midline demarcation of sensory disturbance (see Table 4).

**Table 4:** Midline splitting of light touch and vibration detection

	Motor-FND	Stroke^a^	*P* value^c^
**Light touch**			
Midline splitting of light touch, by patient report	5/102 (4.9%)	5/67 (7.5%)	0.520
Midline splitting of light touch, by clinical examination	2/102 (2.0%)	0	0.519
**Rydel–Seiffer tuning fork vibration detection scores (range 0–8)** ^b^			
Right side of forehead, median (IQR)	7 (5.3, 8)	8 (7, 8)	**<0**.**001**
Number scoring 0	1/101 (1.0%)	2/67 (3.0%)	0.564
Number scoring <6	30/101 (29.7%)	7/67 (10.4%)	0.003
Left side of forehead, median (IQR)	6.7 (5.7, 8)	8 (6.3, 8)	0.011
Number scoring 0	2/101 (2.0%)	2/67 (3.0%)	1.000
Number scoring <6	28/101 (27.7%)	8/67 (11.9%)	0.015
Right side of sternum, median (IQR)	7.5 (6, 8)	8 (7, 8)	**<0**.**001**
Number scoring 0	0	2/67 (3.0%)	0.158
Number scoring <6	22/101 (21.8%)	6/67 (9.0%)	0.029
Left side of sternum, median (IQR)	7.7 (6, 8)	8, (7, 8)	0.004
Number scoring 0	0	2/67 (3.0%)	0.158
Number scoring <6	21/101 (20.8%)	4/67 (6.0%)	0.008
**Rydel–Seiffer tuning fork: frequency of left versus right side difference scores**			
Less than 1 (little or no difference in left versus right vibration perception	47/101 (46.5%)	54/67 (80.6%)	**<0**.**001**
1 or more	55/101 (54.5%)	13/67 (19.4%)	**<0**.**001**
2 or more	33/101 (32.7%)	6/67 (9.0%)	**<0**.**001**
3 or more	16/101 (15.8%)	6/67 (9.0%)	0.195
4 or more	9/101 (8.9%)	2/67 (3.0%)	0.203
5 or more	7/101 (6.9%)	2/67 (3.0%)	0.319
6 or more	5/101 (5.0%)	2/67 (3.0%)	0.704
7 or more	1/101 (1.0%)	2/67 (3.0%)	0.546
8 or more	0	0	
Vibration perception on one side only	4/101 (4.0%)	2/67 (3.0%)	1.000

^a^Eight participants from the stroke group with a positive Hoover’s sign were excluded, as possible cases of FND and stroke.

^b^Rydel–Seiffer tuning fork score ranges from 0 = low/absent vibration detection to 8 = high ability to perceive vibration. Scores of 6 or above were considered normal threshold values for upper limbs in adults under 85 years of age (Martina *et al.* 1998).

^c^Significance testing was performed using chi-square tests or Fisher’s exact test for categorical variables, and Mann–Whitney *U* test for continuous variables. *P*-values that remain significant after Bonferroni correction (threshold 0.05/24 = 0.0021) are shown in bold.

#### Splitting of vibration sense

Vibration detection was tested bilaterally, using a Rydel–Seiffer tuning fork, at the forehead and over the manubrium of the sternum. The mean of three trials was taken, and differences in scores between the left and right sides were considered cases of splitting. Stroke participants with a positive Hoover’s sign were excluded from the data, due to the possibility of stroke presenting with comorbid FND (*n* = 8, amongst which four had splitting of vibration). A one-point or greater left versus right side difference at the forehead and/or sternum, which maps most closely to how clinicians tend to use this test, was found in 55/101 (54.5%) of motor-FND and 13/67 (19.4%) of stroke. This data, including the range of difference thresholds, are reported in Table 4.

The imaging findings of stroke group participants with midline splitting of light touch and/or vibration are displayed in the Supplementary material. Four of the seven cases of splitting of light touch had a stroke territory extending to the thalamus, which is known to cause hemisensory disturbance.^36^ A positive Hoover’s sign was found in 2/7 (28.6%) with splitting of light touch and 4/17 (23.5%) with splitting of vibration sense.

#### Episodes of inconsistency

We asked the motor-FND group about variability of sensory symptoms; 30/98 (31.9%) endorsed experiencing a period of significant improvement or resolution of sensory symptoms, and 1/98 (1.0%) endorsed having a unilateral symptom that swapped to the other side. When asked about the influence of alcohol or recreational drugs on sensory symptoms, 12/68 (17.6%) reported improvement; 53/68 (77.9%) reported no change; and 3/68 (4.4%) reported worsening.

### Quantitative sensory testing (QST)

Overall, we found that QST provided little additional information to the clinical examination. See Supplementary material for the complete MDT, VDT and PPT data.

#### Mechanical detection threshold (MDT)

The spread of MDT classifications was similar for both groups. For example, in the left hand 45.1% of the motor-FND group and 64.0% of the stroke group scored within the normal range, and the spread of scores in categories of diminishing sensation were: ‘diminished light touch’ (27.5% motor-FND versus 13.3% stroke), ‘diminished protective sensation’ (8.8% motor-FND, versus 6.7% stroke), ‘loss of protective sensation’ (11.8% motor-FND versus 5.3 stroke), ‘deep pressure only’ (0% motor-FND versus 0% stroke) and insensate (6.9% motor-FND versus 10.6% stroke).

The agreement between sensory disturbance identified by MDT testing and self-reported sensory symptoms was explored. Agreement was high for the stroke group (left forehead 93%, left hand 81%). Agreement for the motor-FND group was moderate at the forehead (67%) and low in the hands (58%). When there was a lack of agreement, it was more common to have an abnormal MDT in an area that had not been identified as having sensory symptoms (false positive) than a normal MDT in an area reported as having sensory symptoms (false negative). Agreement was low and no better than chance at the feet for both groups. See Supplementary material for full data.

#### Pain pressure threshold (PPT)

Pain pressure thresholds, a measure of tissue sensitivity, were similar for the motor-FND and stroke groups at each of the four sites tested when only females were compared (females are known to have lower PPTs than males^29^). Additionally, the spread of scores was similar between the groups, suggesting there was no clear tendency towards hypersensitivity (25th percentile) or hyposensitivity (75th percentile) in one group compared to the other. See Supplementary material for full data.

### Pain and sensory symptoms

The motor-FND group experienced pain more frequently and more intensively; 89/101 (88.1%) of the motor-FND group reported experiencing regular pain, compared to 30/74 (40.5%) of the stroke group (Table 1). Of interest, more participants in the motor-FND group endorsed perceiving themselves as having a high pain tolerance (70% versus 49%), as measured subjectively by an ordinal scale (‘I have a high pain tolerance’, ‘normal/no different to anyone else’, ‘low pain tolerance’). This tendency in the motor-FND group did not seem to be strongly related to average levels of experienced pain (high tolerance was reported by 68% of those reporting an average pain intensity between 0–5 and 73% reporting average pain intensity between 6 and 10). Body maps of pain distribution differed from the distribution of sensory symptoms, with areas of pain concentrating around proximal joints (see heat maps in Supplementary material).

### Association between sensory symptom severity with clinical outcomes in the motor-FND group

Self-reported sensory symptom severity (7-point ordinal scale) was positively correlated with depression (PHQ-9, Spearman’s rho (*ρ*) = 0.453, *P* < 0.001); anxiety (GAD-7, *ρ* = 0.317, *P* = 0.001); somatic symptom severity (PHQ-15, *ρ* = 0.320, *P* = 0.001); and pain severity (VAS score, *ρ* = 0.279, *P* = 0.005). A significant negative correlation was found with RAND physical functioning (*ρ* = −0.393, *P* < 0.001) and RAND fatigue (*ρ* = −0.369, *P* < 0.001), meaning that greater sensory symptom severity correlated with worse physical disability/fatigue.

These variables were entered into an ordinal regression model to determine which were independently associated with sensory symptom severity. Lower RAND physical functioning (OR 0.98; 95% CI 0.96, 1.00; *P* = 0.031) and higher PHQ-9 depression (OR 1.10; 95% CI 1.00, 1.22; *P* = 0.050) were independent predictors of increased sensory symptom severity. Nagelkerke pseudo *R*^2^ indicated that the model explained 29% of the variance in sensory symptom severity. The proportional odds assumption was met. Pain VAS, GAD-7, RAND fatigue and PHQ-15 were not independently associated with sensory symptom severity in the multivariable model.

### Longitudinal assessment of the motor-FND group

Twelve-month follow-up was completed for 90/102 (88.2%) of the motor-FND group; 27/90 (30.0%) rated their sensory symptoms as having improved, 32/90 (35.6%) reported no change and 31/90 (34.4%) reported worse sensory symptoms (see Table 2, the remaining 12-month data are reported in the Supplementary material). The PHQ-15 (somatic symptom severity) was the only baseline measure that predicted perception of change at 12-months based on ordinal regression modelling. Higher PHQ-15 scores at baseline were associated with worsening perception of sensory symptoms at 12-months (OR 0.89; 95% CI 0.80, 0.99; *P* = 0.038). The proportional odds assumption was met.

We explored the association between improvement in motor symptoms and improved sensory symptoms. In a logistic regression model, participants who had improved motor symptom severity scores at follow-up compared to baseline were twice as likely to report improved sensory symptoms, however this trend was not statistically significant (OR 2.15; 95% CI 0.86, 5.40). In an ordinal regression model, a higher RAND physical functioning score at follow-up increased the odds of reporting an improvement in sensory symptoms at follow-up (OR 1.02; 95% CI 1.00, 1.03; *P* = 0.006), however the effect size is small (Nagelkerke *R*^2^ = 7.8%; proportional odds assumption met).

### Body perceptual disturbance, dissociation and panic at onset

The motor-FND group scored significantly higher than the stroke group for all domains of measurement, exploring potential mechanisms of sensory symptoms. These were body perceptual disturbance, dissociation at symptom onset, current dissociative experiences, and panic at symptom onset. Comparisons were statistically significant surviving a Bonferroni correction for multiple comparisons, except for panic at symptom onset, defined by the proportion reporting four or more panic symptoms, thus meeting DSM 5 TR criteria for a panic attack (*P* = 0.053) see Table 5.

**Table 5:** Potential mechanistic relationships

	Motor-FND, *n* = 102	Stroke, *n* = 75	*P* value^c^
**Revised bath body perception disturbances questionnaire** (score range 0–47)			
Median (IQR)	20.0 (10.0, 26.0)	10.5 (2.0, 20.8)	**<0**.**001**
Number with missing data	7 (6.9%)	3 (4.0%)	
**Peritraumatic dissociative experiences questionnaire** (score range 10–50)			
Median (IQR)	22 (15.5, 27.5)	14 (10.0, 23.0)	**0**.**007**
Number with missing data (only complete answers included)^a^	33/102 (32.4%)	47/75 (62.7%)	
**Dissociative experiences**			
Some people sometimes have the experience of feeling that their body does not belong to them. Circle a number to show what percentage of the time this happens to you: (0%–100%)			
0% of the time, frequency (%)	39/100 (39.0%)	59/74 (79.7%)	**<0**.**001**
10% of the time or more, frequency (%)	61/100 (61.0%)	15/74 (20.3%)	**<0**.**001**
Median % of time (IQR)	20 (0, 60)	0 (0, 0)	**<0**.**001**
**DSM 5 criteria for panic at symptom onset**			
TOTAL number of panic symptoms endorsed, median (IQR)	4 (2, 6)	3 (1,5)	0.033
Number of participants endorsing 4 or more symptoms^b^	48/83 (57.8%)	23/56 (41.1%)	0.053
Symptoms:			
Palpitations/pounding heart	27/99 (27.3%)	11/73 (15.1%)	
Sweating	28/99 (28.3%)	13/73 (17.8%)	
Trembling or shaking	33/99 (33.3%)	16/73 (21.9%)	
Shortness of breath or feeling smothered	25/99 (25.3%)	11/73 (15.1%)	
Feeling of choking	4/99 (4.0%)	3/73 (4.1%)	
Chest pain or discomfort	17/99 (17.2%)	4/73 (5.5%)	
Nausea or abdominal distress	25/99 (25.3%)	12/73 (16.4%)	
Dizzy, unsteady, lightheaded or faint	41/99 (41.4%)	26/73 (35.6%)	
Derealization or depersonalization	35/99 (35.4%)	22/73 (30.1%)	
Feeling of losing control or going crazy	37/99 (37.4%)	15/73 (20.5%)	
Fear of dying	26/99 (26.3%)	17/73 (23.3%)	
Numbness or tingling	52/99 (52.5%)	24/73 (32.9%)	
Chills or hot flushes	29/99 (29.3%)	15/73 (20.5%)	

^a^Participants who were unable to remember onset and answer all questions were excluded from these data. When those with incomplete data were included there was little difference in mean and median scores.

^b^Excluding participants who stated they could not remember. DSM-5 TR criteria for a panic attack require at least 4 of the listed symptoms to be present.

^c^Significance testing was performed using chi-square tests or Fisher’s exact test for categorical variables, and Mann–Whitney *U* test for continuous variables. *P*-values that remain significant after Bonferroni correction (threshold 0.05/7 = 0.007) are shown in bold.

### Exploring the influence of the study assessment on perceptions

Table 3 reports data examining how participating in the study influenced participants’ perceptions of their sensory symptoms. Overall, the majority in both groups reported no change in how they perceived their sensory symptom severity (motor-FND group 66.7% versus stroke group 77.5%). Among those whose opinions changed, 20.2% of motor-FND group and 18.3% of stroke group perceived their sensory symptoms as less severe than they had previously thought, while 13.1% of motor-FND participants and 4.2% of stroke participants felt that their symptoms were worse than initially thought. In response to whether focusing on their symptoms during the testing made them feel worse, a substantially larger proportion of motor-FND group participants (38.7%) reported that this was the case compared to only 7.7% of stroke group participants.

## Discussion

This study explored the frequency, experience and clinical correlates of sensory symptoms amongst a large consecutively recruited cohort of people with motor-FND in comparison to a cohort with acute mild to moderate stroke.

### Frequency and prognosis

We found the frequency of sensory symptoms in motor-FND to be 70% when participants were asked to list their FND symptoms, and 96% after prompting for sensory problems. This was higher than for acute stroke (31% without prompting and 67% after prompting).

Sensory symptoms in FND have been relatively neglected in the modern literature resulting in sparse high-quality comparative data. Recent studies that have reported rates of sensory symptoms range from 64% (based on a clinical interview of 107 people with functional weakness)^37^ to 79% (an online survey of 1048 people with mixed FND symptoms).^3^ Variations are most likely explained by differences in study eligibility criteria, definitions of sensory symptoms and the way questions were framed. In our clinical interviews, we defined sensory symptoms for participants as, ‘… increased or decreased sensitivity or feeling, including problems that may only occur from time to time.’ With regards to stroke, our finding that 67% reported sensory symptoms is consistent with recent studies that found rates of sensory impairment of 57–67% of patients with acute stroke.^38-40^

Our eligibility criteria required motor symptoms as part of the presentation. The frequency and experience of sensory symptoms may differ in people with FND without motor symptoms, such as seizure-dominant presentations and those with only sensory symptoms as the presenting complaint. Isolated hemisensory syndrome is one such presentation. Toth (2003) prospectively identified patients presenting to a tertiary hospital with a hemisensory syndrome; 34 cases were identified and an incidence rate of 2 per 100 000 per year was calculated.^41^ Only one case had abnormal imaging that was thought to be related to the presentation. The problem had resolved in half the patients after 10 days, and for 80% at 16-months follow-up. This is in contrast to our 12-month follow-up data, which found that for 70%, sensory symptoms were either unchanged or worse; 30% reported at least some improvement and only 3% reported their sensory symptoms were very much improved. Isolated sensory symptoms therefore, may have a better prognosis than multi-symptom presentations.

It is notable that 26% of motor-FND and 36% of stroke patients only endorsed experiencing sensory symptoms after prompting. As this occurred in both groups and at a higher frequency in stroke, it is unlikely to be explained only by factors relating to FND. Suggestion and/or expectation resulting from the clinical exam may have contributed to this finding, artificially increasing the endorsement of sensory symptoms, but one might expect this to be more relevant to the motor-FND group. Other factors that may have influenced endorsement of sensory symptoms only after prompting include a low perceived importance, having minimal impact on disability and lack of understanding. The researcher’s perception based on the clinical interview was that only 50% of participants had a clear understanding of the difference between the concepts of weakness and numbness.

### Clinical presentations and notable characteristics

A notable difference between motor-FND and stroke was that FND was associated with a greater variety of altered sensory experiences. The frequency of numbness was relatively similar (68% for motor-FND versus 55% for stroke), but other categories of sensory symptoms were increasingly rare in stroke, for example, paraesthesia (67% versus 20%), abstract experiences (29% versus 5%) and dead or absence type of experiences (20% versus 1%). Other characteristics that were more common in the motor-FND group were sensory symptoms affecting internal surfaces (except inside the mouth), intermittent sensory symptoms, sensory hypersensitivity and having more than one type of sensory symptom. The large variation of abnormal sensory experiences in FND may be a product of multiple causal mechanisms, which we discuss below.

### Impact and significance

Sensory symptoms were considered to be important by people with motor-FND, evidenced by high rates of perceived severity. Over 90% endorsed sensory symptoms to cause some degree of impact on daily life; 33% endorsed sensory symptom severity categories of severe or extreme, which were qualified as sensory symptoms resulting in the need for help from others for daily activities, needing nursing care or hospitalization.

Sensory symptom severity was correlated with other domains of health, including physical disability, depression, anxiety, somatic symptoms severity and pain severity. However, only physical disability and depression were independent predictors of sensory symptom severity, and only 28% of the variance in the severity score was accounted for by these variables. It is intuitive that physical disability/motor symptoms contribute to sensory symptoms as part of a causal mechanism due to the inter-relationship between movement and sensation. This idea is further supported by our finding that sensory and motor symptoms usually co-occurred in limbs. The strong correlation between sensory symptoms severity and other domains of health suggests that sensory symptoms may be a barometer of overall FND severity.

### Clues to understanding mechanisms

The large variation in sensory experiences documented in this study implies multifactorial mechanisms contribute to sensory symptoms in motor-FND. Potential mechanistic explanations are summarized in Table 6, and include the somatosensory consequences of motor symptoms, dissociation, anxiety/panic, co-existing pathology, peripheral circulation dysregulation, sensory processing difficulties (similar to autism spectrum disorder and migraine) and interoceptive inaccuracy. The multifactorial nature relates to the diverse predisposing, precipitating and perpetuating factors that lead to the heterogeneity of clinical presentations of FND.

**Table 6:** Theoretical mechanisms that may contribute to sensory disturbance in FND

**Mechanism: Dual processing of motor and sensory signals** Sensory and motor symptoms may be mutually consequential, representing two sides of the same coin. These two processes are intrinsically linked and processed together within the brain. This idea is fundamental to the theory of predictive processing.^42^ We found that physical disability was independently associated with perceived sensory symptom severity. It would follow that if motor symptoms improved, sensory symptoms would change in tandem. We found some evidence that this relationship existed. The close connection between motor and sensory symptoms may explain why 50% of participants had low confidence in understanding the difference between weakness and numbness.
**Mechanism: Dissociation and compartmentalization** Dissociation includes the experiences of depersonalization and derealization, which describe a feeling of disconnection between the person, their body and/or the environment.^43^ Dissociation has long been considered a mechanistic explanation for symptoms of FND. We found that dissociative experiences were significantly more common in motor-FND compared to stroke,^44^ in both recollections of symptom onset and as part of everyday experiences. The experience of an insensate limb (reported by 27% of motor-FND) and injuries in limbs affected by functional sensory symptoms (reported by 56% of motor-FND) may further be related to the concept of compartmentalization, which is described as a form of dissociation. This concept relates to an inability to bring normally accessible information into conscious awareness or a lack of integration of sensory information within the brain.^45^ Dissociation may be one way in which the brain copes with injury or threat.^46^
**Mechanism: Anxiety, panic and autonomic arousal** The somatosensory consequences of anxiety/panic may contribute to sensory symptoms in FND. The concept of ‘panic without panic’ in FND describes a scenario where people with FND experience symptoms of panic without an associated subjective emotional response.^47^ Without the emotional component, autonomic arousal may be interpreted as symptoms of illness. Participants often described sensory symptoms reminiscent of panic (e.g. ‘electricity moving through my body’), and 51% met the cut-off score for a case of generalized anxiety disorder. However, anxiety was not independently associated with perception of sensory symptom severity. At symptom onset, 58% endorsed experiencing four or more symptoms of panic, thus meeting DSM 5 TR criteria for a panic attack,^20^ although these findings should be interpreted with caution due to recall delay and informal assessment (median 4.4 years). Most of the commonly endorsed symptoms of panic were sensory experiences (e.g. numbness or tingling, dizzy or lightheadedness and chills or hot flushes).
**Mechanism: Co-existing pathophysiology** FND commonly co-exists with other health problems, which may contribute to functional sensory symptoms and often act as predisposing and perpetuating factors for FND onset. Although we excluded people whose symptoms could be explained by other co-existing diagnoses, the sample was typical for FND in that it included people diagnosed with conditions that typically cause sensory symptoms, such as carpal tunnel syndrome and low back pain (with potential radicular symptoms).
**Mechanism: Peripheral vasomotor dysregulation** We had noticed that it was common for people with FND to suffer with cold hands and feet. In the current study, 83% of the motor-FND group compared to 41% of the stroke group endorsed this experience. People with FND also commonly describe dependent oedema and skin discolouration in the hands and feet suggestive of dysregulated peripheral circulation. There may be overlaps with complex regional pain syndrome, which has prominent peripheral vasomotor and temperature features, and both conditions have similar sensory and motor features.^48^
**Mechanism: Sensory processing difficulties, similar to autism spectrum disorder and migraine** Our data shows high rates of self-reported sensory sensitivity (e.g. 65% of motor-FND were hypersensitive to sounds versus 15% of stroke). Sensory sensitivity is common in other neurological conditions, including autism spectrum disorder and migraine, for which there is growing evidence for overlap with FND.^49,50^ These conditions have sensory hypersensitivity and other atypical sensory experiences in common. This suggests shared mechanisms, which have been described using a predictive processing framework. Specifically, there is a failure to attenuate the precision of sensory prediction errors, leading to an amplification or inability to habituate to incoming sensory stimuli.^49,51^ The evidence is speculative.
**Mechanism: Altered interoceptive processing** There is a small body of evidence suggesting interoceptive inaccuracy is associated with FND and specifically sensory symptoms.^47,52,53^ Interoception describes our awareness of the body’s internal state. It can be understood through a predictive processing model, where incoming sensory signals from the body are compared to expectations shaped by our previous experiences and attenuated or amplified.
**Mechanism: Disturbed sense of limb ownership** We observed higher scores on the Bath Body Perception Disturbances Questionnaire in the motor-FND compared to stroke. Although originally developed for complex regional pain syndrome, this scale assesses disturbance in the subjective sense of embodiment, which arises from high-level processing of somatosensory, interoceptive and autonomic stimuli.^15^ Reports of limb absence or a sensation of a ‘dead limb’ in the motor-FND group have parallels at a symptom description level with the disturbed sense of limb ownership described in the stroke literature, particularly in association with right hemisphere lesions.^54^ The phenomenon in stroke is associated with a combination of tactile and proprioceptive deficits, together with altered higher-level cortical processing, including extrapersonal neglect. Future studies could determine if a similar network might be involved in those with this symptom in the context of functional motor symptoms.

We explored the relationship between pain and sensory symptoms. Consistent with the literature, we found that pain was highly prevalent in the motor-FND group.^55^ However, pain severity was not significantly associated with sensory symptom severity after controlling for physical disability and depression. This, together with a finding that pain showed a different distribution from sensory symptoms when mapped on a body chart, suggests that pain and sensory symptoms are more separated in people with FND than some may expect.

### Clinical implications for assessment, diagnosis and treatment

The findings from our study have implications for clinical practice. The ‘classic’ clinical sign of functional sensory symptoms, midline splitting of light touch, lacked sensitivity and specificity for motor-FND when compared to acute stroke, in keeping with recent conclusions in reviews of the topic.^5^ Participants from both groups reported midline splitting of light touch at similar rates. Physical assessment to determine a sharp demarcation of light touch only occurred in the motor-FND group, however at a rate of 2%, it had low clinical utility.

Similarly, splitting of vibration sense occurred in both groups; however, it was more common in motor-FND. A one-point left versus right difference on a Rydel–Seiffer tuning fork at the forehead or sternum occurred in 54% of motor-FND and 19% of acute stroke. Higher threshold difference scores increase the specificity at significant cost to sensitivity (e.g. a four-point or greater difference was found in 9% of motor-FND and 3% of stroke). In clinical practice, vibration splitting is more likely to be tested by asking the patient if vibration on the left feels different from the right side, which may limit the generalizability of this data. Others have also found low specificity for splitting of vibration sense, with reported frequencies in other neurological (non-FND) populations ranging from 11% to 86%.^5^ Despite these limitations, midline splitting of light touch and vibration may still hold value as part of the accumulation of evidence in making a diagnosis of FND. However, our findings suggest there is an unacceptable risk of error if a diagnosis of FND relies on these signs alone.

We found that QST added little information to a typical bedside clinical sensory assessment. Additionally, it led to abnormal findings which were not subjectively reported by the patient. Clinical assessments of people with FND are known to induce transient signs and symptoms which are not specifically associated with disability or distress.^56^ Studies exploring QST in comparable populations have also raised doubts about its clinical utility. A systematic review of detection thresholds in patients with fibromyalgia reported inconsistent findings with no overall significant difference between patients and healthy controls for hyperalgesia and non-noxious tactile stimuli thresholds.^57^

Sensory symptoms were considered an important source of disability by most participants, and therefore, clinicians should ask patients about them and their impact during assessment. Exploring the patient’s understanding of their sensory symptoms may help to expose unhelpful illness beliefs which act as symptom-perpetuating factors (e.g. fearing movements which exacerbate sensory symptoms). Approximately half of our participants did not fully understand the difference between the concepts of numbness and weakness. We therefore suggest taking time to clarify patients' descriptions of their symptoms. Sensory symptoms may also provide a useful model for explaining the diagnosis of FND, as described by Popkirov (2024).^58^

Our data point to there being multiple potential contributors to sensory symptoms in people with motor-FND. These sources may be targeted with multidisciplinary rehabilitation involving physical and psychological therapies. Treatment targets include physical disability, anxiety, depression and dissociation. Addressing these problems may have a corresponding benefit for sensory symptoms. To date there are no effective interventions specifically targeting sensory perception.^5^ Promising avenues of research include interventions aimed at improving interoception,^59^ and occupational therapy for sensory processing difficulties.^60,61^

Current diagnostic criteria for FND in both the International Classification of Diseases (ICD-11)^62^ and the Diagnostic and Statistical Manual of Mental Disorders (DSM 5 TR)^20^ recognize sensory symptoms within the headline definition, and both list ‘sensory loss’ as a diagnostic subtype. We recommend that future iterations continue to recognize sensory symptoms as a core feature of FND, and one that coexists with other symptoms. Additional refinements could include expanding the sensory loss subtype to capture the broader spectrum of sensory symptoms identified in this study, as well as acknowledging the limitations of using sensory signs alone to make a diagnosis. The findings from this study suggest that the symptoms and signs of sensory experiences in FND are, overall, distinct from those of other conditions, although the degree of differentiation is less marked than that observed for functional motor signs or seizure features.

### Limitations

We acknowledge our study has limitations. We report a higher frequency of sensory symptoms compared to previous studies, which may have been influenced by participants being aware of the study topic. Due to the nature of sensory perception, we relied on subjective data from questionnaires and interviews, which are limited by recall bias, validity and reliability issues. Clinical examination may have induced temporary signs and symptoms in the motor-FND group.^56^ Our methods included strategies to minimize suggestion and placebo/nocebo effects, for example, by avoiding leading questions and validating each endorsed experience by asking participants to describe the experience. Our methods of defining categories of sensory symptoms were informed by qualitative research methods and included strategies to validate the findings. The motor-FND group had long symptom durations (median 4.4 years), during which sensory symptoms may evolve and differ from those with acute FND. The groups differed on other clinical and demographic characteristics, including age, sex and ethnicity, which may account for some of the group differences reported in this study. Although our eligibility criteria excluded participants with cognitive impairment and spatial neglect, these problems may have been undetected in some participants, influencing the results. The stroke cohort largely comprised individuals with mild to moderate stroke severity. Including individuals with more severe strokes and spatial neglect might have yielded different results. Additionally, a more thorough and specific assessment of the stroke group may have resulted in different or additional findings. A strength of our study was a large prospectively recruited sample calculated to determine the frequency of sensory symptoms amongst people with motor-FND.

## Conclusion

This prospective observational study found high frequency and diverse sensory symptoms in patients with motor-FND compared to stroke. Sensory symptoms were perceived as being an important contributor to disability and, therefore, should be considered in clinical assessments. Furthermore, sensory symptoms remained unchanged or deteriorated over a 12-month follow-up for most participants, mirroring prognosis data for motor symptoms.^63^ The diverse range of experiences suggests that sensory symptoms relate to multiple contributing factors, which may be targeted with multidisciplinary rehabilitation. Further research should explore the potential for specific interventions to target troublesome sensory symptoms and their potential role as predictive biomarkers of outcome.

## Supplementary Material

fcag031_Supplementary_Data

## Data Availability

Deidentified participant data can be made available by request to the corresponding author. Requests will be considered after planned analyses and reporting have been completed by the investigators. Access will require submission of a protocol that is approved by a review committee.
