# Supplementary material for: Functional sensory symptoms and signs: a case-control study of 102 patients
Source: Brain Commun. 2026 Feb 3;8(1):fcag031. doi: 10.1093/braincomms/fcag031 (PMC12909258; doi:10.1093/braincomms/fcag031)
Supplement: fcag031_Supplementary_Data [file fcag031_supplementary_data.pdf]

Functional sensory symptoms and signs:
A case control study of 102 patients

Supplementary Materials

Table of Contents

Materials and Methods 2
Outcome Measures 2
Mechanical Detection Threshold Assessment Procedure 4
Vibration Detection Threshold Assessment Procedure 6
Pain Pressure Threshold Testing Procedure 8
Results 9
Other potential sources of sensory disturbance 9
Stroke group: lesion location 10
Categorisation of sensory descriptors 11
Imaging reports of stroke participants with midline split of light touch 14
Imaging reports of stroke participants with midline split of vibration sense 15
Mechanical Detection Threshold 16
Vibration Detection Threshold 23
Pain Pressure Threshold 26
Sensory Symptom and Pain Heat Maps 29
Longitudinal Assessment Data, baseline and 12-month follow-up for the motor-FND group 30

Materials and Methods

Outcome Measures

Sensory Symptom Severity

Over the last week, the severity of my **sensory** symptoms
(feelings of numbness, pins & needles, loss of sensation/sensitivity)
has been (please tick one box):

	1. No symptoms	I don't experience this symptom
	2. Borderline	Symptoms have had a subtle or very minor impact
	3. Mild	Symptoms have had a mild impact, causing problems occasionally
	4. Moderate	Symptoms have had a moderate impact, causing difficulties at least a few days in the week
	5. Marked	Symptoms have interfered with social/work/school activity or have been distressing most days of the week
	6. Severe	Symptoms have been very disruptive every day and you require help from others for your usual activities
	7. Extreme	Symptoms interfere with most daily activities and you require help from others for daily activities and you have needed hospitalisation or nursing care to cope

Motor Symptom Severity

Over the last week, the severity of my **motor** symptoms
(abnormal movement, including problems with walking and using your arms)
Has been (please tick one box):

	1. No symptoms	I don't experience this symptom
	2. Borderline	Symptoms have had a subtle or very minor impact
	3. Mild	Symptoms have had a mild impact, causing problems occasionally
	4. Moderate	Symptoms have had a moderate impact, causing difficulties at least a few days in the week
	5. Marked	Symptoms have interfered with social/work/school activity or have been distressing most days of the week
	6. Severe	Symptoms have been very disruptive every day and you require help from others for your usual activities
	7. Extreme	Symptoms interfere with most daily activities and you require help from others for daily activities and you have needed hospitalisation or nursing care to cope

Self-Rated Change in Sensory Symptom Severity at follow-up

Clinical Global Impression Scale of Change
Compared to 1 year ago, my sensory symptoms (numbness, pins and needles or sensory loss) are:

- ☐ Very much improved
- ☐ Much improved
- ☐ Minimally improved
- ☐ No change
- ☐ Minimally worse
- ☐ Much worse
- ☐ Very much worse

Mechanical Detection Threshold Assessment Procedure

Mechanical Detection Thresholds were assessed using Semmes-Weinstein Monofilaments (SWM), The Touch-Test^{TM}, North Coast Medical. The testing kit contained 20 monofilaments, which ranged in applied force from 0.008 grams to 300 grams.

SWM have been found to have good reliability and validity in a stroke population, testing at the tips of the thumb and index finger (Suda et al 2021).

The testing procedure was informed by the equipment instruction manual, as well as published recommendations (Rolke et al 2006, Baumgärtner et al 2002). Thresholds were determined using method of limits approach.

- Starting at the lowest pressure filament, the examiner (author RH) applied the filament perpendicular to the skin until the filament bowed and held it in place for approximately 1.5 seconds. The filament is applied 3 times to elicit a response. Ascending filament pressures are applied until the participant is able to detect the stimulus (suprathreshold).
- Next, starting from the filament pressure above the suprathreshold, filaments are applied in descending order until the participant is unable to perceive the stimulus (infrathreshold).
- The process is repeated 5 times, each time recording a supra- and infra-threshold.
- The final value is the geometric mean of the 5 infra- and 5-supra threshold values.

Supplementary Figure 1. Mechanical detection threshold testing sites (both sides were tested)

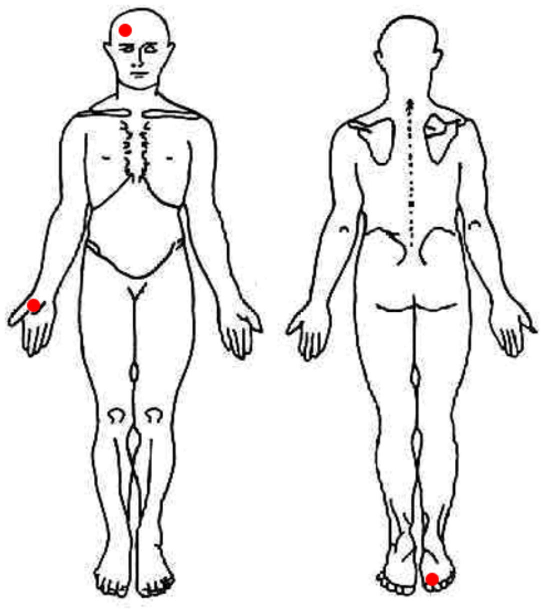

Head	Lateral forehead, directly above the midline of the eye
Hand	Centre of thenar eminence
Foot	Plantar surface over the head of first metatarsal

Supplementary References:

Baumgärtner U, Magerl W, Klein T, Hopf HC, Treede RD. Neurogenic hyperalgesia versus painful hypoalgesia: Two distinct mechanisms of neuropathic pain. Pain. 2002;96(1–2):141–51.

Rolke R, Magerl W, Campbell KA, Schalber C, Caspari S, Birklein F, et al. Quantitative sensory testing: A comprehensive protocol for clinical trials. Eur J Pain. 2006;10(1):77–88.

Suda M, Kawakami M, Okuyama K, Ishii R, Oshima O, Hijikata N, et al. Validity and Reliability of the Semmes-Weinstein Monofilament Test and the Thumb Localizing Test in Patients With Stroke. Front Neurol. 2021; 11:625917.

The results are presented as frequencies for published threshold categories, which have been found to be valid and reliable in a stroke population (Hooper and Ruettermann 2022, Suda et al 2021)

Touch-Test™ Sensory Evaluator Chart. The 20-piece Semmes-Weinstein monofilament kit, evaluator label, force applied be evaluator in grams and category of sensory impairment for hands and plantar surface of the foot.

<table>
<tr><th></th><th>Evaluator label</th><th>Target force (grams)</th><th>Hand and dorsal foot thresholds</th><th>Planter thresholds</th></tr>
<tr><td>1</td><td>1.65</td><td>0.008</td><td rowspan="4">Normal</td><td rowspan="6">Normal</td></tr>
<tr><td>2</td><td>2.36</td><td>0.02</td></tr>
<tr><td>3</td><td>2.44</td><td>0.04</td></tr>
<tr><td>4</td><td>2.83</td><td>0.07</td></tr>
<tr><td>5</td><td>3.22</td><td>0.16</td><td rowspan="2">Diminished light touch</td></tr>
<tr><td>6</td><td>3.61</td><td>0.4</td></tr>
<tr><td>7</td><td>3.84</td><td>0.6</td><td rowspan="4">Diminished protective sensation</td><td rowspan="4">Diminished light touch</td></tr>
<tr><td>8</td><td>4.08</td><td>1</td></tr>
<tr><td>9</td><td>4.17</td><td>1.4</td></tr>
<tr><td>10</td><td>4.31</td><td>2</td></tr>
<tr><td>11</td><td>4.56</td><td>4</td><td rowspan="9">Loss of protective sensation</td><td rowspan="3">Diminished protective sensation</td></tr>
<tr><td>12</td><td>4.74</td><td>6</td></tr>
<tr><td>13</td><td>4.93</td><td>8</td></tr>
<tr><td>14</td><td>5.07</td><td>10</td><td rowspan="6">Loss of protective sensation</td></tr>
<tr><td>15</td><td>5.18</td><td>15</td></tr>
<tr><td>16</td><td>5.46</td><td>26</td></tr>
<tr><td>17</td><td>5.88</td><td>60</td></tr>
<tr><td>18</td><td>6.10</td><td>100</td></tr>
<tr><td>19</td><td>6.45</td><td>180</td></tr>
<tr><td>20</td><td>6.65</td><td>300</td><td>Deep pressure sensation only</td><td>Deep pressure sensation only</td></tr>
</table>

Supplementary References:

Hooper G, Ruettermann M. Reporting numerical values for sensory testing. J Hand Surg Eur Vol. 2022;47(11):1178–80.

Suda M, Kawakami M, Okuyama K, Ishii R, Oshima O, Hijikata N, et al. Validity and Reliability of the Semmes-Weinstein Monofilament Test and the Thumb Localizing Test in Patients With Stroke. Front Neurol. 2021; 11:625917.

Vibration Detection Threshold Assessment Procedure

A Rydel-Seiffer (RS) tuning fork was used to measure vibration detection thresholds (VDT). The RS tuning fork vibrates at 64 Hz and has an arbitrary ordinal scale from 0-8, which is a measure of vibration intensity. The score is taken from when the vibration is no longer perceived (Martina et al 1998).

RS Scale (Supplementary Figure 3). As the fork vibrates, the perceived intersection of the triangles moves in an exponential fashion from 0 (low/absent vibration detection) to 8 (high ability to perceive vibration) with decreasing vibration amplitude (figure adapted from Martina et al 1998).

Supplementary Figure 2. A graphical illustration of the Rydel-Seiffer tuning fork ordinal scale, ranging from 0-8. The intersection of the triangles moves as vibration amplitude reduces.

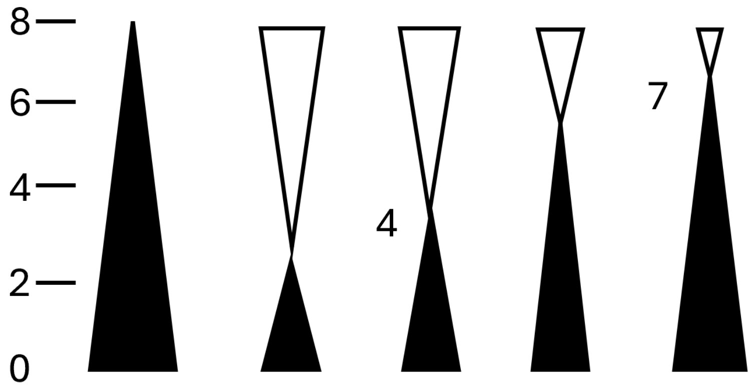

The RS tuning fork has been found to have good intra- and inter- observer agreement and acceptable responsiveness to change (Merkies et al 2000).

The tuning fork was applied over a bony prominence, perpendicular to the body segment. The participant was asked to indicate when they no longer perceived the decreasing vibration stimulus. The mean of 3 tests were taken.

It is reported that there is decreasing vibration sensitivity with age (Martina 1998). Martina et al reported normal thresholds (5% lower limit) as follows:

</= 40 yrs	upper limbs >/= 6.5	</= 40	lower limbs >/= 4.5
41-85 yrs	upper limbs >/= 6.0	41-60	lower limbs >/= 4.0
> 85 yrs	upper limbs >/= 5.5	61-85	lower limbs >/= 3.5
		>85	lower limbs >/= 3.0

Supplementary References:

Martina ISJ, Van Koningsveld R, Schmitz PIM, Van Der Meché FGA, Van Doorn PA. Measuring vibration threshold with a graduated tuning fork in normal aging and in patients with polyneuropathy. J Neurol Neurosurg Psychiatry. 1998;65(5):743–7.

Merkies ISJ, Schmitz PIM, Van Der Meché FGA, Van Doorn PA. Reliability and responsiveness of a graduated tuning fork in immune mediated polyneuropathies. The Inflammatory Neuropathy Cause and Treatment (INCAT) Group. J Neurol Neurosurg Psychiatry. 2000; 68(5):669–71.

Supplementary Figure 3. Testing sites for vibration detection threshold testing (both sides were tested)

<table>
<tr><td rowspan="4">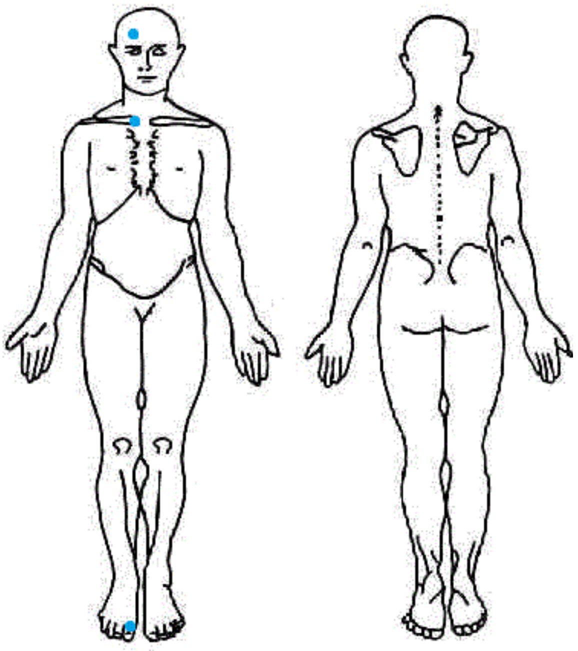</td><td>Head</td><td>Lateral forehad, above the midline off the eye</td></tr>
<tr><td>Trunk</td><td>Lateral edge of the manubrium</td></tr>
<tr><td>Foot</td><td>First toe, bony prominance of the tarsometatarsal joint</td></tr>
<tr><td></td><td></td></tr>
</table>

Patient position
Sitting on a chair. The measurement is taken at the point the patient no longer perceives vibration.

The examiner demonstrated on an intact area the feeling of vibration and no vibration. Tests were conducted bilaterally, starting at asymptomatic side (or right), starting at the head.

Pain Pressure Threshold Testing Procedure

A pressure algometer was used to determine pain pressure thresholds, Wagner Instruments Pain Test^{TM}. Thresholds were collected following the device instruction manual.

The participant was instructed “Tell me the moment when pressure changes to slightly unpleasant pain. This means the very first onset of discomfort or pain and not the most pressure that you can bear”.

The algometer was held perpendicular to the body and force was applied at a rate of roughly five N/second. The body segment being tested was supported. Tests were conducted bilaterally, starting at asymptomatic side (or right), moving distally. Test units N/cm^2. The mean of three tests were taken.

The maximum applied pressure was 100N. If the participant had fragile skin or there appeared to be risk of injury, testing pressure was stopped at 50N.

Supplementary Figure 4. Testing sites for pain pressure threshold testing (both sides were tested)

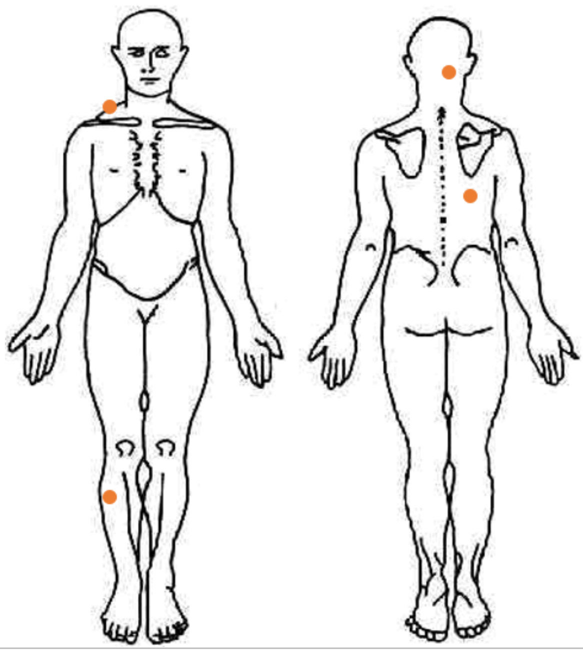	Suboccipital	Below the occipatal bone, lateral to the trapezius muscle insertion.
	Shouder	Upper fibres of trapezius, at the midopint between C7 spinous process and the lateral acromium.
	Mid Thoracic	4cm below the inferior angle of the scapula.
	Leg	Muscle belly of tibialis anterior, approx. 2.5 cm lateral and 5 cm distal to the tibial tubercle.

Results

Other potential sources of sensory disturbance

Supplementary Table 1. Other potential sources of sensory disturbance

	FND		Stroke	
**Other possible sources of sensory disturbance**	**n**	**%**	**n**	**%**
None identified	85	85.0	56	80.8
Bowel adhesions	1	1.0	-	
Burning mouth syndrome	1	1.0	-	
Carpal tunnel syndrome (history of or current)	4	4.0	2	2.7
Chiari malformation	2	2.0	-	
Diabetes (type 1 or 2)	2	2.0	6	8.2
Gout	-		1	1.4
Lymphoedema	-		1	1.4
Meralgia	1	1.0	2	2.7
Orthopaedic injury (current)[a]	1	1.0	1	1.4
Raynaud's phenomenon	2	2.0	-	
Thyroid cancer / tongue cancer	1	1.0	1	1.4
Vaginal prolapse	1	1.0	-	
TOTAL	100	100.0	73	100.0
Missing data	2		2	

For the purposes of this study, Fibromyalgia was considered part of the spectrum of FND and not considered an alternative cause of sensory disturbance.
a: Orthopaedic injuries were ankle sprain (FND) and left shoulder fracture (Stroke).

Stroke group: lesion location

Supplementary Table 2. Anatomical locations of strokes, extracted from MRI or CT imaging reports

Mechanism	Anatomical Location	Left	Right	Bilateral	Total
**Haemorrhage**	Parietal	1			1
	Frontoparietal	2			2
	Temporoparietal		1		1
	Insula		1		1
	Basal ganglia	2	2		4
	Thalamus	2			2
	Internal capsule	1			1
	Pons			1	1
**Infarct**	**Vascular territory**				
	Anterior Cerebral Artery	2	1		3
	Middle Cerebral Artery	6	15		21
	Posterior Cerebral Artery	3	3		6
	**Cortical Regions**				
	Frontal		1		1
	Parietal lobe		1		1
	Postcentral gyrus		1		1
	Insula		2		2
	Parietal and anterior limb of the internal capsule			1	1
	Occipitotemporal infarct with petechial hemorrhages		1		1
	Occipital and cerebellar		1		1
	**Subcortical Regions**				
	Basal ganglia		1	1	2
	Thalamic		1		1
	Corona radiata (with or without extension)	3	2		5
	Lenticulostriate	3	3		6
	Lacunar infarct (not otherwise specified)	2			2
	Striatocapsular and lentiform nucleus		1		1
	Thalamocapsular	1			1
	Brainstem			2	2
	Pontine		1		1
	Medulla	2	1		3
	**TOTAL**	**30**	**40**	**5**	**75**

Categorisation of sensory descriptors

Categorisation of sensory descriptions followed an inductive qualitative process, informed by Elo and Kyngäs 2008. [Elo S, Kyngäs H. The qualitative content analysis process. J Adv Nurs. 2008;62(1):107–15.]

Process
1. Descriptions of sensory symptoms were extracted from the interviews
    - Motor-FND group: n=277 from 96 participants with sensory symptoms.
    - Stroke group: n=77 from 50 participants with sensory symptoms
2. Two researchers (RH and GN) reviewed the descriptions independently before engaging in the consensus process to group them into categories. Cross-references were made to the original audio-recorded interviews to provide context and clarity when needed and to resolve differences in interpretation.
3. An initial set of 14 categories was created and iteratively cross-checked against the source data to ensure the categories were grounded in participants' accounts.
4. The preliminary categories were then discussed with the wider team (GN, RH, MJE, JS, JC) in a reflexive process, which resulted in condensing the 14 categories into 11.
5. The categories were then further condensed into the final six overarching categories of sensory experiences in the FND and stroke groups.

Supplementary Table 3. Six categories were derived from a longer list of 11 categories

6 Categories		11 Categories	
1	Numb/reduced	1	Numb/reduced
2	Paraesthesia	2	Paraesthesia
3	Dead/absent	3	Dead/absent
4	Muscle related	4	Muscle related
5	Abstract (subsumed the categories below)	5	Abstract
	Tight/pressure	6	Tight/pressure
	Thermal regulation	7	Thermal regulation
	Pulsatile	8	Pulsatile
	Travelling	9	Travelling
	Internal vibration/electricity	10	Internal vibration/electricity
6	Insensate / no sensation	11	Non-specific
	Non-specific – "I don't know" / "different"		

Supplementary Table 4. Grouping into 11 Categories, confirmed by reapplying to the participants' descriptions

		FND, N=96		Stroke, N=50	
		n	%	n	%
1	Numb/reduced	69	71.9%	41	82.0%
2	Paraesthesia	68	70.8%	15	30.0%
3	Dead/absent	20	20.8%	1	2.0%
4	Abstract positive	8	8.3%	2	4.0%
5	Tight/pressure	9	9.4%	1	2.0%
6	Thermal regulation	2	2.1%	1	2.0%
7	Muscle related	11	11.5%	2	4.0%
8	Pulsatile	3	3.1%	0	0.0%
9	Travelling	2	2.1%	0	0.0%
10	Non-specific	2	2.1%	3	6.0%
11	Internal vibration/electricity	6	6.3%	0	0.0%
	2. Paraesthesia subtypes	FND, N=65		Stroke, N=15	

a	Pins and needles	30	46.2%	5	33.3%
b	Hypersensitivity	15	23.1%	4	26.7%
c	Other positive	28	43.1%	7	46.7%
d	Burning / hot	8	12.3%	1	6.7%
e	Cold tingling	7	10.8%	0	0.0%
g	Electricity	4	6.2%	0	0.0%

Supplementary Table 5. Utterances derived from participants' descriptions were grouped into preliminary categories. These were refined and combined into 11 categories, which were subsequently condensed into six overarching categories.

FND		Stroke	
**Numb**	**54**	**Numb**	**36**
**Pins & needles**	**30**	**Pins & needles**	**6**
**Other positive phenomena**	**54**	**Other positive phenomena**	**7**
Tingling, 27		Tingling, 6	
Electricity, 9		Tingles when touched, 1	
Buzzing, 3			
Crawling, 3			
Bubbling, 2			
Vibration, 2			
Water dripping/wet, 2			
Fizzing, 1			
Humming, 1			
Internal tremor, 1			
Nervy, 1			
Static, 1			
Wiggly worm, 1			
**Dead / absent / not there**	**37**	**Dead / absent / not there**	**3**
Not there, 10		Belong to someone else, 1	
Heavy, 7		Hallucination, 1	
Dead / dead weight, 6		Heavy, 1	
Detached / disintegrated / empty body, 4			
Not mine / prosthetic, 3			
Blobby / sponge, 2			
Hanging on by a tendon, 1			
Hollow bones, 1			
No knees, 1			
Tube replaced femur, 1			
Turned to putty, 1			
**Reduced**	**28**	**Reduced**	**12**
Loss, 10		Loss, 5	
Reduced / dull / muted / less, 9		No sensation, 2	
Buffer / plastic film / hard skin / thick covering, 4		Reduced, 2	
Fuzzy, 2		Cardboard b/n fingers, 1	
Fluffy, 1		Lack of precision, 1	
Loss sensitivity during sex, 1		Fingers feel like marshmallows, 1	
Paper, 1			

FND			Stroke	
**Hypersensitivity / prickly / itchy**	**16**		**Hypersensitivity**	**3**
Hypersensitive, 7			Hypersensitive, 1	1
Itch, 2			Jolt, 1	1
Prickly, 2			Bruised, 1	
Insect bite, 1				
Pinching, 1				
Rash, 1				
Hair on face / cobwebs, 1				
Grit in eye, 1				
**Burning / hot / fire**	**12**		**Hot**	**1**
**Tight, pressure**	**10**		**Tight**	**1**
Pressure / swollen / tight / tight covering, 7			Elastic band, 1	
Fluid filled, 1				
Elastic band, 1				
Big, 1				
**Cold/temp related**	**9**		**Cold/temp related,**	**3**
Cold / ice, 6			Cold, 2	
Cold tingling / frozen but warm, 2			Can’t tell hot from cold, 1	
Temperature sensitivity, 1				
**Muscle related**	**6**			
Weak, 2				
Tired muscles, 1				
Tension, 1				
Stiffening, 1				
Pulled, 1				
**Travelling**	**5**			
Cold travelling, 2				
Travelling, 1				
Shooting, 1				
Waves, 1				
**Pulsatile**	**5**			
Pulsing / body tinnitus, 3				
Palpitations, 1				
Thudding, 1				
**Other / cross categories and were qualifiers**	**7**		**Other**	**8**
Altered, 1			Different, 2	
Brain related, 2			Don’t know, 1	
Dry mouth, 1			*Feels like it is moving, 1	
Glue on surface, 1			Funny, 1	
Switched on, 1				
Variable, 1				

Imaging reports of stroke participants with midline split of light touch

Supplementary Table 6. Imaging reports of stroke participants with midline split of light touch

**ID**	**Splitting based on subjective patient report**	**Sharp demarcation on clinical exam**	**FND signs found on exam**	**Imaging report**
2027	Full midline split of temperature perception, reduced on the right, that is most noticeable in the shower with warm water. Was uncertain how sharp the demarcation was.	No	No	MRI: Acute infarct posterolateral in the medulla oblongata on the left. The remainder of the intracranial appearances are unremarkable.
2036	Described midline, reduced on the left, that became progressively duller laterally.	No	No	CT: Right posterior cerebral artery infarct. No acute intracranial haemorrhage.
2045*	Full midline split, described as "numbness" on the left side.	No	Hoover's sign and hip abductor sign on left side.	MRI: A tiny acute lacunar infarct is noted in the right centrum semiovale. A possible but not definite small cortical infarct is also noted within the right middle frontal gyrus.
2046	Described complete right hemi-sensory loss with a midline demarcation.	No	No	CT: Small volume intraparenchymal haemorrhage centred on the left **thalamus** with involvement of the posterior limb of the internal capsule.
2054	The right side of the body felt "blurry".	No	No	MRI: There is evolution of the haematoma in the left **thalamocapsular** region.
2059*	Numbness on the right side of trunk/body.	Yes	Hoover's sign on right side.	CT: Left **thalamocapsular** haematoma with mild surrounding oedema.
2060	Hemibody sensory loss on the right side.	No	No	MRI: There are several small acute infarcts in the left PCA territory including the left hippocampus, left geniculate region, left **thalamus**, left perisplenial and a couple small foci in the left medial parieto-occipital region.

*Had a positive Hoover's sign, therefore excluded from the count of stroke group participants with midline splitting of light touch due to possible dual diagnosis of FND, as described in the manuscript.

Imaging reports of stroke participants with midline split of vibration sense

Supplementary Table 7. Imaging reports of stroke participants with midline split of vibration sense

ID	Rydel-Seiffer tuning fork, left vs right side difference score	Stroke type and territory as described in the official report
2002	1	MRI: Left ACA infarct. Incidental finding of mature cortical infarct within right occipital lobe around calcarine fissure
2008*	3	MRI: Tiny focus of diffuse abnormality in the right posterior temporal lobe with may be an acute infract.
2011	3	CT: Right subacute anterior cerebral artery infarct
2012*	6	CT: Left sided posterior cerebral artery infarct
2033	1	CT: Left corona radiata infarcts
2036	7	CT: Right PCA infarct
2043	3	CT: Right MCA infarct
2045*	6	MRI: tiny acute lacunar infarct in the right centrum semiovale
2046	3	CT: Left **thalamic** ICH
2047	1	CT: Multifocal right temporoparietal ICH
2049	1	CT: Bilateral basal ganglia lacunar infarcts
2060	7	CT: Left **thalamus** ICH
2061	1	CT: Left hemispheric lacunar infarct
2071	1	CT: Right MCA infarct with haemorrhagic transformation
2074	1	MRI: Left PCA infarcts
2054	3	MRI: Right M1 occlusion with right MCA infarct
2082*	4	MRI: Left **thalamocapsular** infarct

*Had a positive Hoover's sign, therefore excluded from the count of stroke group participants with midline splitting of vibration due to possible dual diagnosis of FND, as described in the manuscript.

Mechanical Detection Threshold

Supplementary Table 8. MDT Threshold Categories: FND vs Stroke

	FND n, %		STROKE n, %	
**Forehead, right**				
Normal	78	76.5%	69	92.0%
Diminished light touch	10	9.8%	3	4.0%
Diminished protective sensation	6	5.9%	0	0.0%
Loss of protective sensation	7	6.9%	0	0.0%
Deep pressure only	0	0.0%	0	0.0%
Unable to feel	1	1.0%	3	4.0%
**Forehead, left**				
Normal	77	75.5%	70	93.3%
Diminished light touch	9	8.8%	2	2.7%
Diminished protective sensation	8	7.8%	0	0.0%
Loss of protective sensation	7	6.9%	0	0.0%
Deep pressure only	1	1.0%	0	0.0%
Unable to feel	0	0.0%	3	4.0%
**Hand, right**				
Normal	44	43.1%	60	80.0%
Diminished light touch	34	33.3%	5	6.7%
Diminished protective sensation	11	10.8%	1	1.3%
Loss of protective sensation	7	6.9%	3	4.0%
Deep pressure only	1	1.0%	3	4.0%
Unable to feel	5	4.9%	3	4.0%
**Hand, left**				
Normal	46	45.1%	48	64.0%
Diminished light touch	28	27.5%	10	13.3%
Diminished protective sensation	9	8.8%	5	6.7%
Loss of protective sensation	12	11.8%	4	5.3%
Deep pressure only	0	0.0%	0	0.0%
Unable to feel	7	6.9%	8	10.6%
**Foot, right**				
Normal	46	45.1%	31	41.3%
Diminished light touch	23	22.5%	14	18.7%
Diminished protective sensation	8	7.8%	8	10.7%
Loss of protective sensation	7	6.9%	8	10.7%
Deep pressure only	3	2.9%	1	1.3%
Unable to feel	15	14.7%	13	17.3%
**Foot, left**				
Normal	41	40.2%	24	32.0%
Diminished light touch	22	21.6%	16	21.3%
Diminished protective sensation	8	7.8%	7	9.3%
Loss of protective sensation	8	7.8%	11	14.7%
Deep pressure only	0	0.0%	4	5.3%
Unable to feel	23	22.5%	13	17.3%

Plots of MDT applicator label (left) and threshold category frequency (right)

Supplementary Figure 5. Mechanical detection thresholds (MDT). Dot plots for applicator frequency (left column) and category of sensory disturbance (right column).
A: Right side of forehead; B: Left side of forehead; C: Right hand; D: Left hand; E: Right foot; F: Left foot.

MDT Applicator Label

Dot plots of geometric mean of evaluator label (applicator size). This data is indicative only, as the applicator size is rated on a logarithmic scale.

MDT Threshold Category

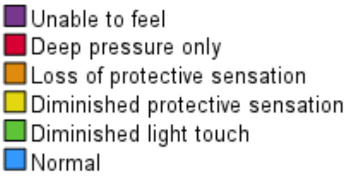

A

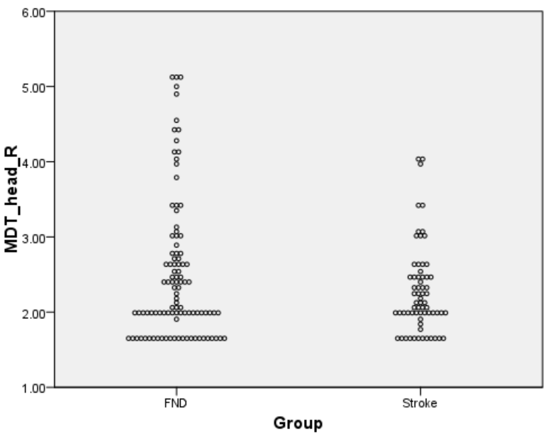

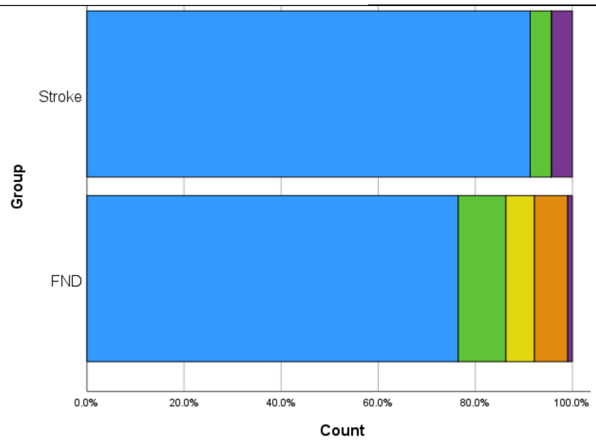

B

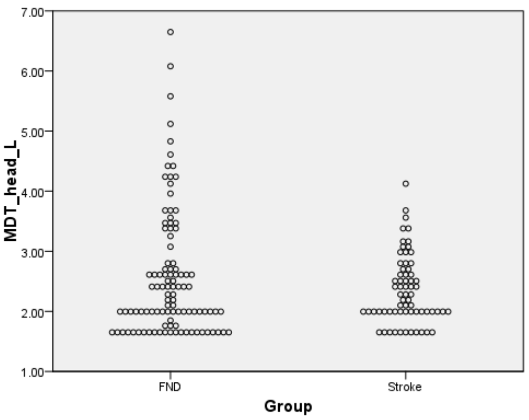

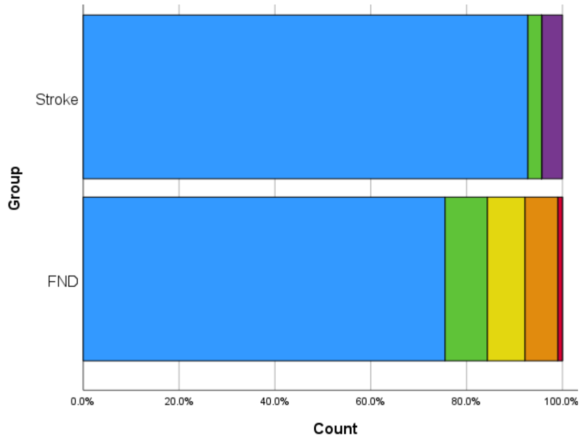

C

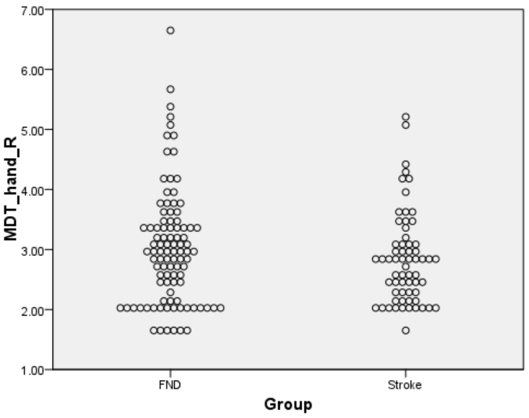

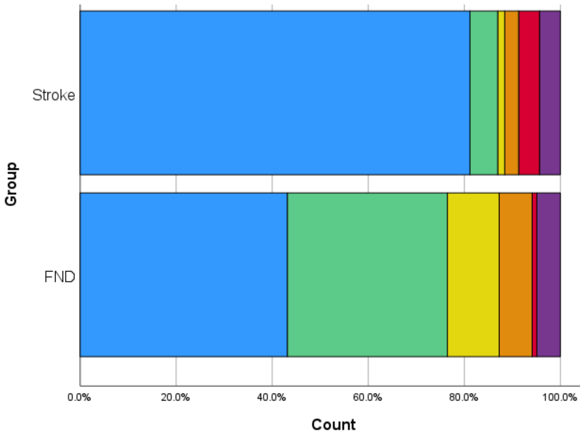

<table>
<tr><th></th><th>MDT Applicator Label</th><th>MDT Threshold Category</th></tr>
<tr><td></td><td>Dot plots of geometric mean of evaluator label (applicator size). This data is indicative only, as the applicator size is rated on a logarithmic scale.</td><td>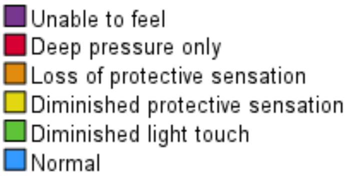
</td></tr>
<tr><td>D</td><td>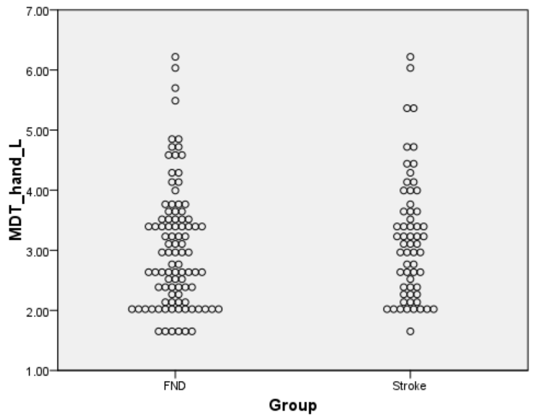
</td><td>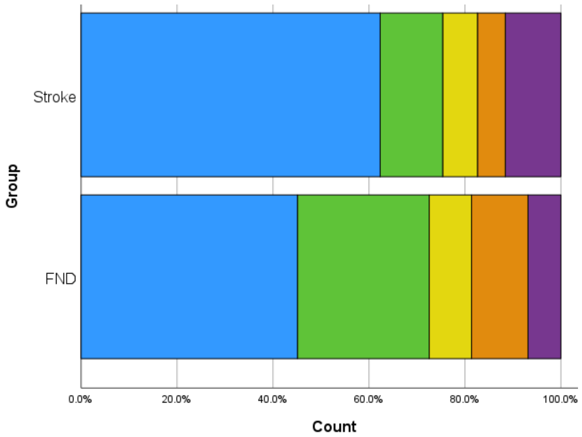
</td></tr>
<tr><td>E</td><td>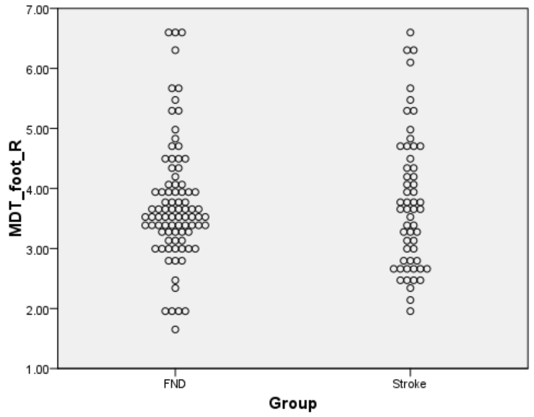
</td><td>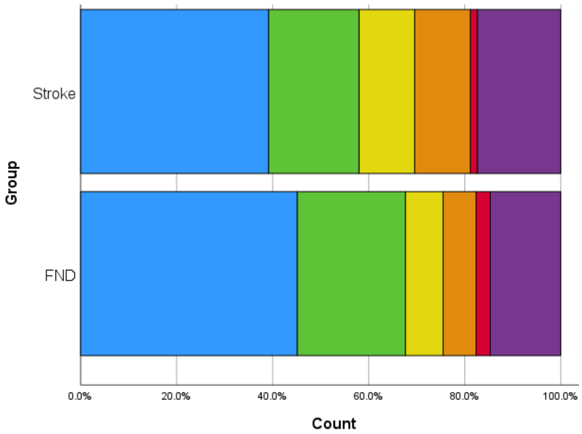
</td></tr>
<tr><td>F</td><td>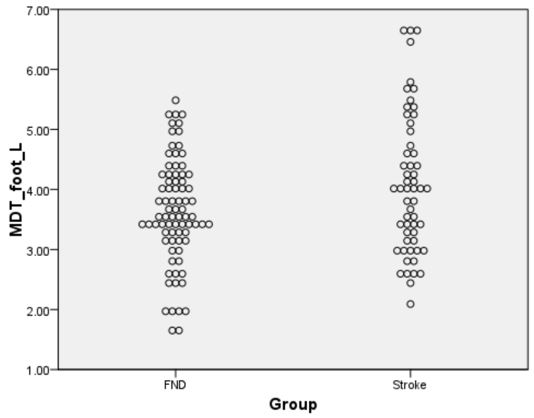
</td><td>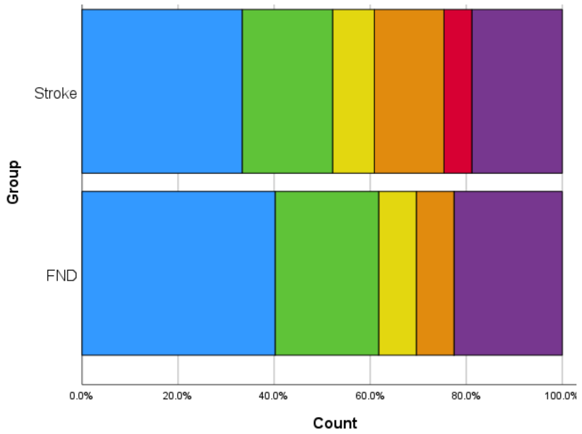
</td></tr>
</table>

MDT Force in grams (supra-thresholds)

The supra-threshold was tested 5 times, or until two consecutive responses of the same value were obtained. The modal value is was taken as the threshold for each participant and reported below is the group median and mode force for supra-thresholds. Note that where participants who were unable to report a threshold due to an absence of sensation, it is not represented in this data.

Supplementary Table 9. MDT Fource in grams (supra-thresholds)

		**FND supra-threshold force in grams (applicator size)**	**Stroke supra-threshold force in grams (applicator size)**
Forehead Right	Median	0.04 (2.44)	0.04 (2.44)
	Mode	0.02 (2.36)	0.02 (2.36)
Forehead Left	Median	0.04 (2.44)	0.04 (2.44)
	Mode	0.008 (1.65)	0.02 (2.36)
Hand Right	Median	0.16 (3.22)*	0.07 (2.83)
	Mode	0.4 (3.61)*	0.04 (2.44)
Hand Left	Median	0.16 (3.22)*	0.16 (3.22)*
	Mode	0.4 (3.61)*	0.4 (3.61)*
Foot Right	Median	0.6 (3.84)*	0.6 (3.84)*
	Mode	0.4 (3.61)	0.07 (2.83)
Foot Left	Median	0.6 (3.84)*	1.4 (4.17)*
	Mode	0.4 (3.61)	0.4 (3.61)

*outside of normal range

Comparison of abnormal MDT with self-reported areas of sensory symptoms

MDT testing site did not always correspond to areas of reported sensory loss. Here we compare normal vs abnormal MDT threshold with areas of sensory disturbance reported by participants on the body map.

"False negatives" were test results where the MDT was recorded as normal in an area that the participant reported as having sensory symptoms on the body map. "False positives" were tests results where MDT was recorded as abnormal in an area that was not reported as having sensory symptoms on the body map. These are not necessarily "false" results as the MDT finding may be a true representation of the sensory function and the participant may have forgotten to report the sensory symptom or may have been unaware of the sensory disturbance.

Supplementary Table 10. Comparison of abnormal MDT with self-reported areas of sensory symptoms

Testing Site	% reporting sensory disturbance	% with abnormal MDT	MDT "True positives"	MDT "True negatives"	MDT "False negative"	MDT "False positive"	Congruent "correctly classified"
**FND group, mechanical detection threshold scores in the abnormal range**							
Forehead, R	17/100 (17.0%)	23/100 (23.0%)	3 (3.0%)	63 (63.0%)	14 (14.0%)	20 (20.0%)	**66.0%**
Forehead, L	20/100 (20.0%)	23/100 (23.0%)	5 (5.0%)	62 (62.0%)	15 (15.0%)	18 (18.0%)	**67.0%**
Hand, R	38/100 (38.0%)	56/100 (56.0%)	24 (24.0%)	30 (30.0%)	14 (14.0%)	32 (32.0%)	**54.0%**
Hand, L	41/100 (41.0%)	55/100 (55.0%)	27 (27.0%)	31 (31.0%)	14 (14.0%)	28 (28.0%)	**58.0%**
Foot, R	33/100 (33.0%)	54/100 (54.0%)	19 (19.0%)	32 (32.0%)	14 (14.0%)	35 (35.0%)	**51.0%**
Foot, L	39/100 (39.0%)	60/100 (60.0%)	22 (22.0%)	23 (23.0%)	17 (17.0%)	38 (38.0%)	**45.0%**
**Stroke group, mechanical detection threshold scores in the abnormal range**							
Forehead, R	6/75 (8.0%)	6/75 (8.0%)	4 (5.3%)	67 (89.3%)	2 (2.7%)	2 (2.7%)	**94.7%**
Forehead, L	6/75 (8.0%)	5/75 (6.7%)	3 (4.0%)	67 (89.3%)	3 (4.0%)	2 (2.7%)	**93.3%**
Hand, R	14/75 (18.7%)	15/75 (20.0%)	8 (10.7%)	54 (72.0%)	6 (8.0%)	7 (9.3%)	**82.7%**
Hand, L	19/75 (25.3%)	27/75 (36.0%)	16 (21.3%)	45 (60.0%)	3 (4.0%)	11 (14.7%)	**81.3%**
Foot, R	14/75 (18.7%)	44/75 (58.7%)	13 (17.3%)	30 (40.0%)	1 (1.3%)	31 (41.3%)	**57.3%**
Foot, L	19/75 (25.3%)	51/75 (68.0%)	15 (20.0%)	20 (26.7%)	4 (5.3%)	36 (48.0%)	**46.7%**

Chi square tests were conducted to test for an association between reported sensory disturbance as drawn on a body map and abnormal MDT. The results can be found below.

Supplementary Table 11. Chi square tests of association between reported sensory disturbance as drawn on a body map and abnormal MDT

FND Group

FND: Right forehead Chi Square

	Normal MDT right forehead	Abnormal MDT right forehead
Normal sensation reported	63	20
Sensory disturbance reported	14	3

Assumptions for chi squared are violated as one cell has an expected count of <5. Therefore we should use Fisher's Exact test. **p=0.755**. No association between mapped sensory disturbance on the right side of the forehead and abnormal MDT was found.

FND: Left forehead Chi Square

	Normal MDT left forehead	Abnormal MDT left forehead
Normal sensation reported	62	18
Sensory disturbance reported	15	5

Assumptions for chi squared are violated as one cell has an expected count of <5. Therefore we should use Fisher's Exact test. **p=1.0**. No association was found between mapped sensory disturbance on the left side of the forehead with abnormal MDT.

FND: Right hand Chi Square

	Normal MDT	Abnormal MDT
Normal sensation reported	30	32
Sensory disturbance reported	14	24

Assumption met. X^2 (1) = 1.274, **p=0.303** No association was found between reported sensory disturbance in the right hand with abnormal MDT.

FND: Left hand Chi Square

	Normal MDT	Abnormal MDT
Normal sensation reported	31	28
Sensory disturbance reported	14	27

Assumption met. X^2 (1) = 3.308, **p=0.102**. No association was found between mapped sensory disturbance of the left hand with abnormal MDT

FND: Right foot Chi Square

	Normal MDT	Abnormal MDT
Normal sensation reported	32	35
Sensory disturbance reported	14	19

Assumption met. X^2 (1) = 0.254, **p=0.673**. No association was found between mapped sensory disturbance of the right foot with abnormal MDT.

FND: Left foot Chi Square

	Normal MDT	Abnormal MDT
Normal sensation reported	23	38
Sensory disturbance reported	17	22

Assumption met. X^2 (1) = 0.343, **p=0.676**. No association was found between mapped sensory disturbance of the left foot with abnormal MDT.

When tests were repeated with only FND participants who described negative sensory disturbance (numbness, reduced, sensory loss etc.), there were no significant associations found between areas or reported sensory loss and abnormal MDT findings. There are fewer false negatives, but no consistent improvement to the number correctly classified.

Supplementary Table 12. Chi square tests of association between reported sensory disturbance as drawn on a body map and abnormal MDT

Stroke Group

Stroke: Right forehead Chi Square

	Normal MDT right forehead	Abnormal MDT right forehead
Normal sensation reported	62	2
Sensory disturbance reported	1	4

Assumptions for chi squared are violated as at least one cell has an expected count of <5. Therefore we should use Fisher's Exact test. **p<0.001**. There is a statistically significant association between sensory disturbance reported on the body map and corresponding MDT.

Stroke: Left forehead Chi Square

	Normal MDT left forehead	Abnormal MDT left forehead
Normal sensation reported	61	2
Sensory disturbance reported	3	3

Assumptions for chi squared are violated as at least one cell has an expected count of <5. Therefore we should use Fisher's Exact test. **p=0.004**. There is a statistically significant association between sensory disturbance on the body map and corresponding MDT.

Stroke: Right hand Chi Square

	Normal MDT	Abnormal MDT
Normal sensation reported	50	6
Sensory disturbance reported	6	7

Assumptions for chi squared are violated as at least one cell has an expected count of <5. Therefore we should use Fisher's Exact test. **p=0.002**. There is a statistically significant association between sensory disturbance on the body map and corresponding MDT.

Stroke: Left hand Chi Square

	Normal MDT	Abnormal MDT
Normal sensation reported	41	11
Sensory disturbance reported	2	15

Assumptions met. X^2 (1) = 24.55, **p<0.001**. There was an association between mapped sensory disturbance and MDT.

Stroke: Right foot Chi Square

	Normal MDT	Abnormal MDT
Normal sensation on map	26	30
Sensory disturbance on map	1	12

Assumptions met. X^2 (1) = 6.65, **p=0.01**. there was an association.

Stroke: Left foot Chi Square

	Normal MDT	Abnormal MDT
Normal sensation on map	19	32
Sensory disturbance on map	4	14

Assumption met. X^2 (1) = 1.35, **p=0.245**. No association was found between

Unlike the FND group, there was a statistically significant association between MDT classification and areas reported to be affected by sensory symptoms on the body map for all areas, except the left foot. False positives (abnormal tests in areas not reported as having sensory symptoms) disrupted the association.

Vibration Detection Threshold

Supplementary Table 13. Rydel-Seiffer tuning fork scores

Scores range from 0=low/absent vibration detection to 8=high ability to perceive vibration.

	**FND**	**Stroke**
Forehead, right	N=102	N=75
Mean (SD)	6.4 (1.8)	7.2 (1.1)
Median (IQR)	7 (5.3, 8.0)	8 (7, 8)
Min	0.7	0
Max	8	8
Unable to feel (%)	1 (1.0%)	2 (2.7%)
Forehead, left	N=101	N=75
Mean (SD)	6.6 (1.6)	7.2 (1.4)
Median (IQR)	7 (5.7, 8)	8 (6.3, 8)
Min	1.3	0
Max	8	8
Unable to feel (%)	2 (2.0%)	2 (2.7%)
Sternum, right	N=100	N=75
Mean (SD)	6.7 (1.6)	7.4 (1.1)
Median (IQR)	7.5 (6, 8)	8 (7, 8)
Min	1	2
Max	8	8
Unable to feel (%)	0	2 (2.7%)
Sternum, left	N=100	N=75
Mean (SD)	6.8 (1.5)	7.4 (1.2)
Median (IQR)	7.7 (6, 8)	8 (7, 8)
Min	2	0
Max	8	8
Unable to feel (%)	0	2 (2.7%)
Foot, right	N=101	N=62
Mean (SD)	6.7 (1.5)	5.6 (2.4)
Median (IQR)	8 (6, 8)	6 (4.6, 8)
Min	0	0
Max	8	8
Unable to feel (%)	10 (9.9%)	8 (10.7%)
Foot, left	N=102	N=59
Mean (SD)	6.4 (2.4)	5.1 (2.3)
Median (IQR)	8 (5.9, 8)	5.3 (4, 7)
Min	0	0
Max	8	8
Unable to feel (%)	12 (11.8%)	10 (13.3%)

Plots

Supplementary Figure 6. Dot plots of vibration detection thresholds (VDT).
Each dot represents the recorded VDT, measured on an arbitrary ordinal scale from 0-8 (y-axis). Panels: A: Right side of forehead; B: Left side of forehead; C: Right side of sternum; D: Left side of sternum: E: Right foot; F: Left foot.

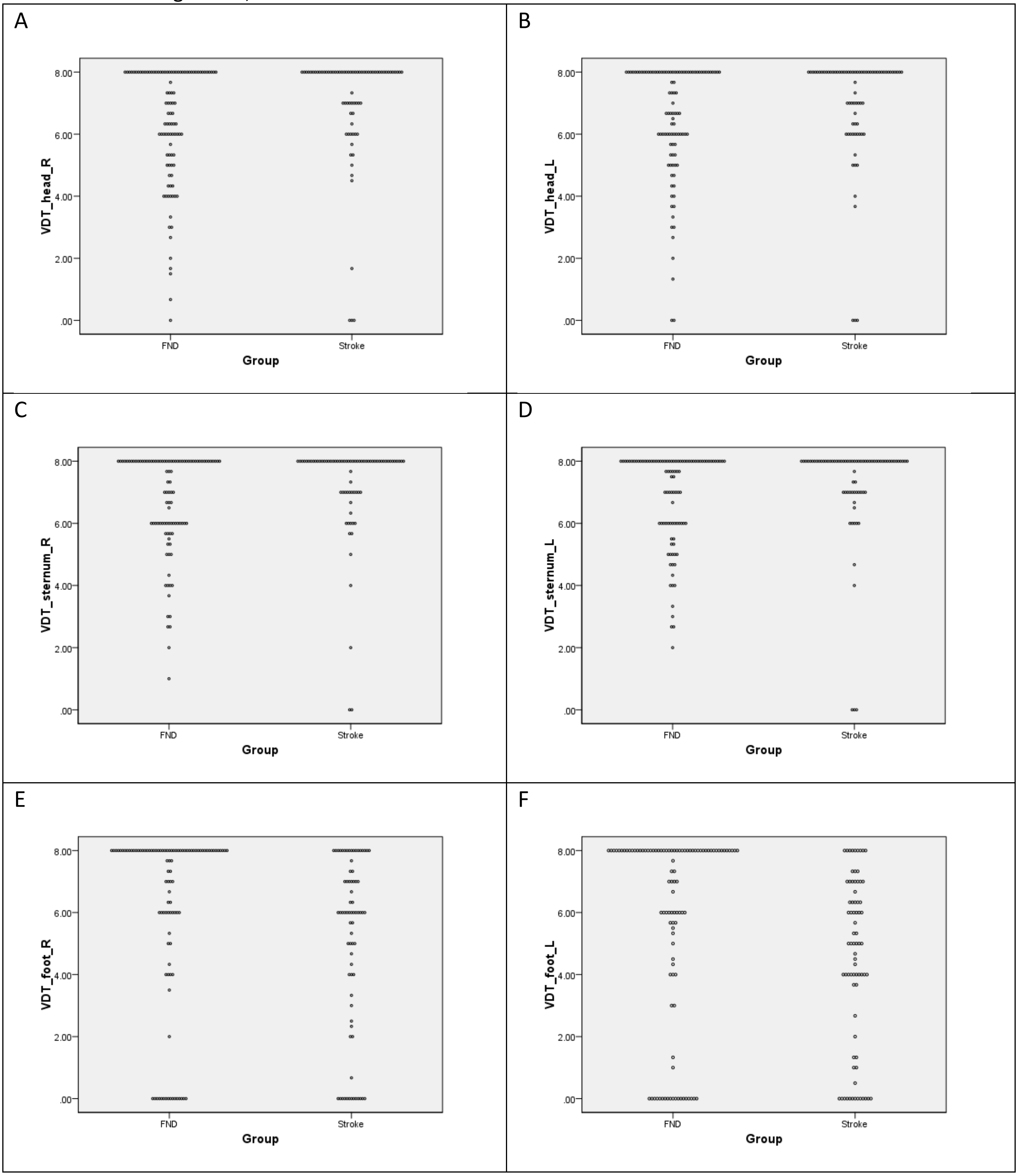

Thresholds

Normal threshold (5% lower limit) (Martina et al 1998):

</= 40	upper limbs >/= 6.5	</= 40	lower limbs >/= 4.5
41-85	upper limbs >/= 6.0	41-60	lower limbs >/= 4.0
> 85	upper limbs >/= 5.5	61-85	lower limbs >/= 3.5
		>85	lower limbs >/= 3.0

In the FND group, n=48 were under 40 years old; n=54 were older than 40; n=1 was older than 85.
In the stroke group, n=2 participants were 40 years old or younger; n=4 were older than 85.

Supplementary Table 14. Thresholds (normal/abnormal) for vibration detection
head and sternum: VDT, 6 or above = normal; less than 6 = abnormal
Foot: VDT 3.5 and above = normal, less than 3.5 abnormal (conservative choice); a threshold of 3.0 was considered for participants older than 85 years.

	FND	Stroke	Chi-Square
Forehead, right	N=102	N=74	
Normal	72 (70.6%)	64 (86.5%)	
Abnormal	30 (29.4%)	10 (13.5%)	
			p=0.013
Forehead, left	N=101	N=75	
Normal	74 (73.3%)	66 (88.0%)	
Abnormal	27 (26.7%)	9 (12.0%)	
			p=0.012
Sternum, right	N=100	N=75	
Normal	78 (78.0%)	68 (90.7%)	
Abnormal	22 (22.0%)	7 (9.3%)	
			p=0.026
Sternum, left	N=100	N=75	
Normal	79 (79.0%)	70 (93.3%)	
Abnormal	21 (21.0%)	5 (6.7%)	
			p=0.008
Foot, right	N=101	N=75	
Normal	84 (83.2%)	55 (73.3%)	
Abnormal	17 (16.8%)	20 (26.7%)	
			p=0.149
Foot, left	N=102	N=75	
Normal	79 (77.5%)	55 (73.3%)	
Abnormal	23 (22.5%)	20 (26.7%)	
			p=0.528

Supplementary Reference:

Martina ISJ, Van Koningsveld R, Schmitz PIM, Van Der Meché FGA, Van Doorn PA. Measuring vibration threshold with a graduated tuning fork in normal aging and in patients with polyneuropathy. J Neurol Neurosurg Psychiatry. 1998;65(5):743–7.

Pain Pressure Threshold

Supplementary Table 15. Pain pressure thresholds, comparing only female participants. Units are N/cm^2. 95% CI calculated using boot strapping with 1000 repetitions.

	**FND**	**Stroke**
**Suboccipital, right**	N=79	N=36
Mean (SD)	11.9 (7.7)	13.4 (9.4)
25 (95% CI)	6.4 (5.2, 8.4)	7.2 (5.4, 8.9)
**50 (95% CI)**	**10.4 (9.1, 12.4)**	**10.8 (8.2, 14.1)**
75 (95% CI)	15.6 (13.3, 19.1)	15.2 (13.5, 23.2)
Max pressure reached	N=1	N=0
**Shoulder, right**	N=78	N=36
Mean (SD)	20.7 (10.6)	19.6 (10.5)
25 (95% CI)	12.8 (10.1, 15.6)	10.4 (7.6, 14.0)
**50 (95% CI)**	**20.0 (16.7, 22.1)**	**18.3 (12.7, 25.8)**
75 (95% CI)	27.3 (22.7, 30.9)	27.5 (25.1, 31.1)
Max pressure reached	N=1	N=0
**Mid Thoracic, right**	N=75	N=36
Mean (SD)	24.8 (14.8)	20.9 (11.0)
25 (95% CI)	13.8 (10.2, 17.3)	11.4 (8.1, 17.7)
**50 (95% CI)**	**21.8 (17.8, 25.8)**	**19.2 (15.5, 24.3)**
75 (95% CI)	36.3 (31.5, 40.7)	28.8 (20.9, 35.3)
Max pressure reached	N=4	N=0
**Leg, right**	N=68	N=36
Mean (SD)	30.3 (17.4)	28.8 (13.1)
25 (95% CI)	15.4 (10.8, 23.2)	20.1 (10.2, 26.1)
**50 (95% CI)**	**29.3 (26.8, 35.1)**	**27.7 (23.8, 35.7)**
75 (95% CI)	39.7 (35.5, 46.7)	40.3 (31.7, 43.9)
Max pressure reached	N=11 (16%)	N=3 (8.3%)

Supplementary Figure 7. Pain pressure threshold (PPT) dot plots.

Each dot represents the recorded PPT, measured in units N/cm^2 (y-axis).

Panels: A: Right suboccipital; B: Left suboccipital; C: Right shoulder; D: Left shoulder; E: Right mid-thoracic; F: Left mid-thoracic; G: Right leg; H: Left leg.

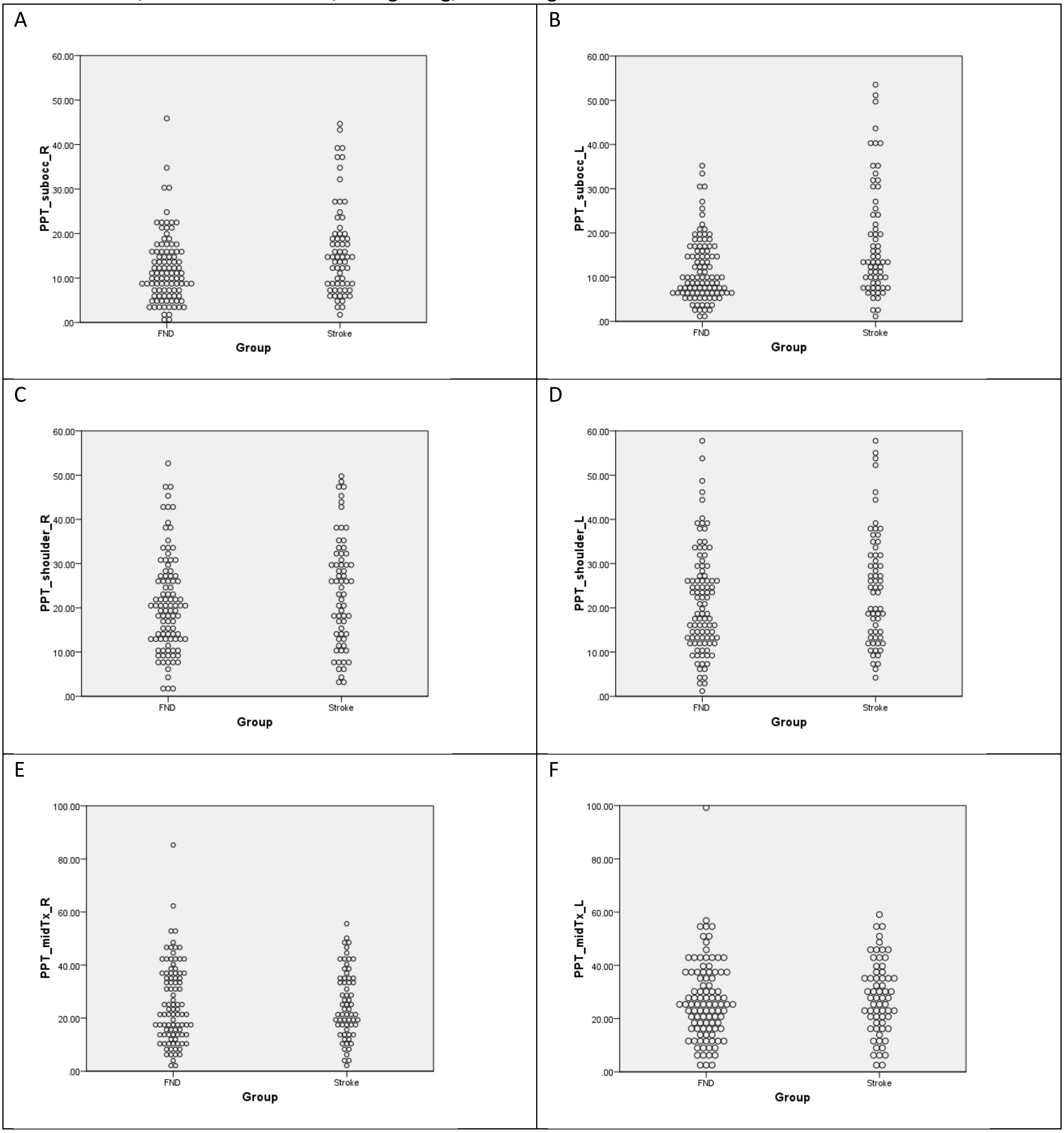

G

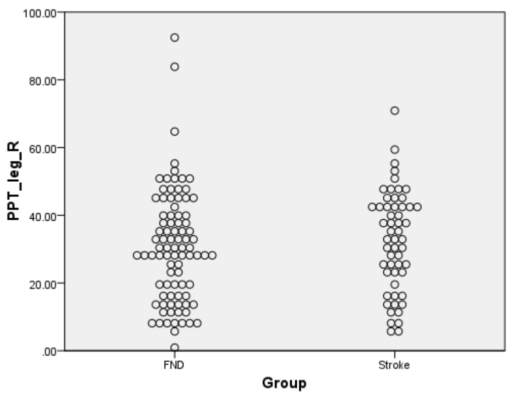
100.00
80.00
60.00
40.00
20.00
.00
PPT_leg_R
FND
Stroke
Group

H

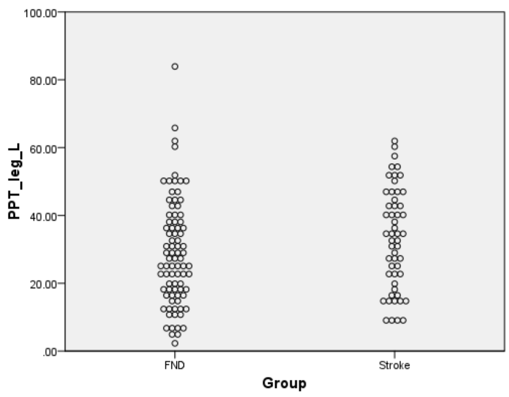
100.00
80.00
60.00
40.00
20.00
.00
PPT_leg_L
FND
Stroke
Group

Sensory Symptom and Pain Heat Maps

Supplementary Figure 8. Heat-maps of the distribution of sensory symptoms (teal shades) and pain (red shades) as a percentage of the sample, for the motor-FND and stroke groups.
Panels: A: Heat maps for areas of reported sensory symptoms, motor-FND and stroke groups; B: Heat maps for areas of reported pain, motor-FND and stroke groups.

A

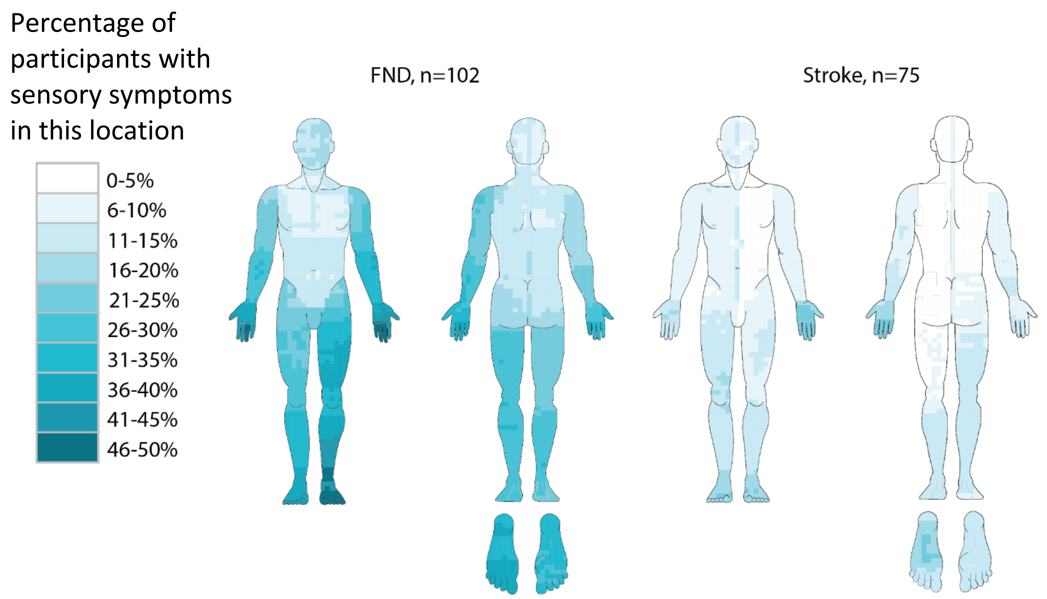

B

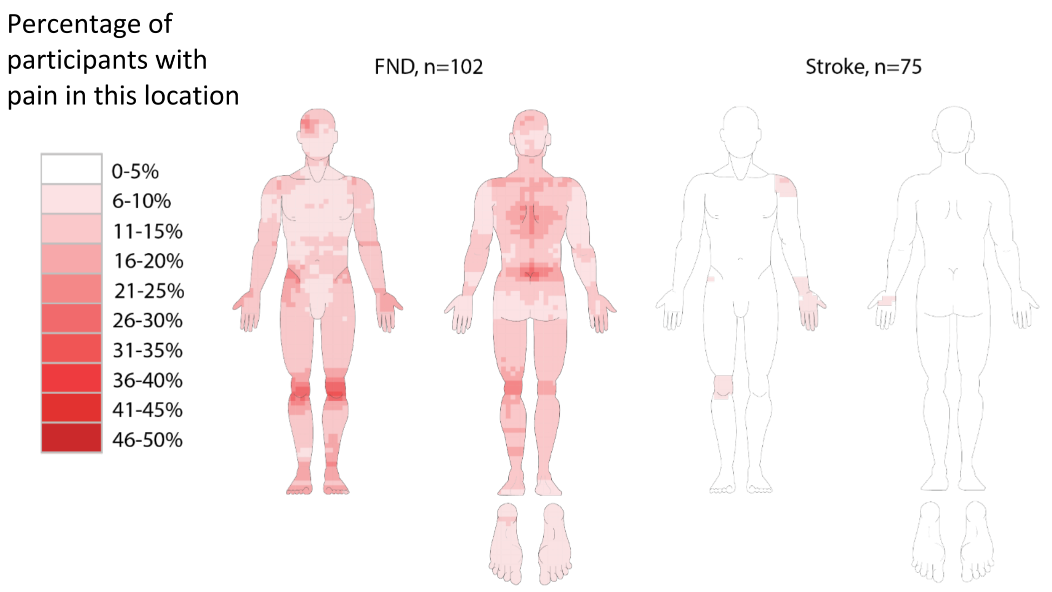

Longitudinal Assessment Data, baseline and 12-month follow-up for the motor-FND group

Supplementary Table 16. Longitudinal Assessment Data, baseline and 12-month follow-up for the motor-FND group

	Baseline	12-month follow-up
**Number included (%)**	Max 102	Max 90 (88.2%)
**Mean time to follow-up in days**		364.7 (SD 17.4)
**Domains from the RAND 36-Item Health Survey**		
Physical Functioning (score range 0-100), mean (SD)	35.7 (20.8)	38.0 (27.6)
Energy/Fatigue domain (score range 0-100), mean (SD)	26.2 (20.7)	28.1 (20.4)
**Functional Mobility Scale**		
Mean total score /18 (SD)	11.9 (4.9)	12.0 (4.7)
Mobility aid needed over 50m		
Independent	18 (18.0%)	16/89 (18.0%)
Independent but rails for stairs	31 (31.0%)	26/89 (29.2%)
Walking stick(s)	17 (17.0%)	20/89 (22.5%)
Crutches	11 (11.0%)	8/89 (9.0%)
Walker	5 (5.0%)	5/89 (5.6%)
Wheelchair	18 (18.0%)	14/89 (15.7%)
**Patient Health Questionnaire-9** (PHQ-9, score range 0-27)		
Mean (SD)	12.8 (6.4)	12.5 (6.1)
N scoring 10 or above (cut-off score for cases of depression)	69/101 (68.3%)	60/89 (67.4%)
**Generalised Anxiety Disorder-7** (GAD-7, score range 0-21)		
Mean (SD)	9.5 (5.9)	9.7 (6.0)
N scoring 10 or above (cut-off score for cases of anxiety)	51/101 (50.5%)	43/89 (48.3%)
**Patient Health Questionnaire-15** (PHQ-15, score range 0-30)		
Mean (SD)	12.3 (4.8)	12.6 (5.8)
**Self-rated Quality of Life**		
Very good	6 (6.0%)	8/88 (9.1%)
Good	18 (18.0%)	13/88 (14.8%)
Neither good nor poor	46 (46.0%)	39/88 (44.3%)
Poor	22 (22.0%)	20/88 (22.7%)
Very poor	8 (8.0%)	8/88 (9.1%)
**Pain intensity over the last week** (VAS 0-10)		
Mean (SD)	5.2 (2.7)	5.9 (2.5)
Median (IQR)	5 (4, 7)	6 (4, 8)
**Confidence in correctness of diagnosis of FND** (0-10)		
Mean (SD)	8.4 (2.3)	8.0 (2.5)
Median (IQR)	9 (8, 10)	
**Motor Symptom Severity** (7-point ordinal scale)		
No motor symptoms [a]	2/101 (2.0%)	3/90 (3.3%)
Borderline (subtle or very minor impact)	3/101 (3.0%)	3/90 (3.3%)
Mild (causing problems occasionally)	5/101 (5.0%)	10/90 (11.1%)
Moderate (causing difficulties a few days in the week)	22/101 (21.8%)	27/90 (30.0%)
Marked (interfered with social/school/work most days)	24/101 (23.8%)	16/90 17.8%)
Severe (result in needing help from others for daily activity)	32/101 (31.7%)	22/90 (24.4%)

Extreme (need hospitalization or nursing care to help)	13/101 (12.9%)	9/90 (10.0%)
**Sensory Symptom Severity** (7-point ordinal scale)		
No sensory symptoms	4/101 (4.0%)	3/90 (3.3%)
Borderline (subtle or very minor impact)	5/101 (5.0%)	3/90 (3.3%)
Mild (causing problems occasionally)	19/101 (18.8%)	18/90 (20.0%)
Moderate (causing difficulties a few days in the week)	25/101 (24.8%)	27/90 (30.0%)
Marked (interfered with social/school/work most days)	15/101 (14.9%)	13/90 (14.4%)
Severe (result in needing help from others for daily activity)	23/101 (22.8%)	17/90 (18.9%)
Extreme (need hospitalization or nursing care to help)	10/101 (9.9%)	9/90 (10.0%)
**Clinical Global Impression Scale of Change at 12-months** Compared to 1 year ago, my sensory symptoms (numbness, tingling, pins and needles, etc.) are		
Very much worse		7/90 (7.8%)
Much worse		7/90 (7.8%)
Minimally worse		17/90 (18.9%)
No change		32/90 (35.6%)
Minimally improved		16/90 (17.8%)
Much improved		8/90 (8.9%)
Very much improved		3/90 (3.3%)
Total reporting improvement		63/90 (70.0%)
Total reporting no change or worse		27/90 (30.0%)
